# Supplementary material for: Identifying critical growth stage and resilient genotypes in cowpea under drought stress contributes to enhancing crop tolerance for improvement and adaptation in Cameroon
Source: PLoS One. 2024 Jun 28;19(6):e0304674. doi: 10.1371/journal.pone.0304674 (PMC11213307; doi:10.1371/journal.pone.0304674)
Supplement: Attachment — Submitted filename: PONE-D-23-38326_reviewer.pdf [file pone.0304674.s001.pdf]

PLOS ONE

Yield-based drought tolerance indices in cowpea exposed to stress at vegetative and flowering setting stages identifies sensitive growth phase and drought tolerant genotypes in prospect to crop improvement

--Manuscript Draft--

Manuscript Number:	PONE-D-23-38326
Article Type:	Research Article
Full Title:	Yield-based drought tolerance indices in cowpea exposed to stress at vegetative and flowering setting stages identifies sensitive growth phase and drought tolerant genotypes in prospect to crop improvement
Short Title:	Yield-based drought tolerance indices in cowpea identifies sensitive stage and drought tolerant genotypes
Corresponding Author:	Eric Bertrand Kouam<br>University of Dschang: Universite de Dschang<br>Dschang, CAMEROON
Keywords:	Key worlds: Cowpea genotypes; Drought stress; Drought tolerance; Growth stage; Selection indices
Abstract:	Drought stress following climate change is likely a scenario that will have to face crop growers in tropical regions. In mitigating this constraint, the best option should the selection and use of resilient varieties that can withstand drought threats. Therefore, a pot experiment was conducted under greenhouse conditions at the Research and Teaching Farm of the Faculty of Agronomy and Agricultural Sciences of the University of Dschang, with the objectives of identifying sensitive growth stage and drought-tolerant genotypes following vegetative and early flowering induced stresses, with the help of yield-based selection indices. Eighty-eight cowpea genotypes from the sahelian and western regions of Cameroon were subjected to drought stress at vegetative (VDS) and flowering setting (FDS) stages by withholding water for 28 days, using a split plot design with two factors and three replications. Seed yields under stress (Ys) and non-stress (Yp) conditions were recorded. Fifteen drought indices were calculated for the two drought stress levels against the yield from non-stress plants. Drought intensity indice (DII) under VDS and FDS were 0.71 and 0.84 respectively, indicating severe drought stress for both stages. However, flowering setting stage was significantly more sensitive to drought stress compared to vegetative stage. Based on PCA and correlation analysis, the indices STI, REI, GMP, MP, YI and HM correlated strongly with yield under stress and non-stress conditions and are therefore suitable to discriminate high-yielding and tolerant genotypes under both stress and non-stress conditions. Either under VDS and FDS, CP-016 exhibited an outstanding performance under drought stress and was revealed as the most drought tolerant genotype as shown by ranking, PCA and cluster analysis. Taking into account all indices, the top five genotypes namely CP 016, CP 021, MTA22, CP 056 and CP060 were identified as the most drought-tolerant genotypes under VDS. For stress activated at flowering setting stage (FDS), CP-016, CP-056, CP-021, CP-028 and MTA-22 were the top five most drought-tolerant genotypes. Several genotypes with valueless Ys and rank among which CP-037, NDT-001, CP036, CP-034, NDT-002, CP-031, NDT-011 were identified as highly drought sensitive with low yield stability. This study identified the most sensitive stage and drought tolerant genotypes that are proposed for genetic improvement of cowpea.
Order of Authors:	Toscani Ngompe-Deffo
	Eric Bertrand Kouam
	Marie Solange Mamdou
	Raba Allah-To Bara
	Asafor Henry Chotangui
	Adamou Souleymanou

Powered by Editorial Manager® and ProduXion Manager® from Aries Systems Corporation

	Honore Beyegue-Djonko
**Additional Information:**	
**Question**	**Response**
**Financial Disclosure**<br><br>Enter a financial disclosure statement that describes the sources of funding for the work included in this submission. Review the submission guidelines for detailed requirements. View published research articles from *PLOS ONE* for specific examples.<br><br>This statement is required for submission and **will appear in the published article** if the submission is accepted. Please make sure it is accurate.<br><br>**Funded studies**<br>Enter a statement with the following details:<br>• Initials of the authors who received each award<br>• Grant numbers awarded to each author<br>• The full name of each funder<br>• URL of each funder website<br>• Did the sponsors or funders play any role in the study design, data collection and analysis, decision to publish, or preparation of the manuscript?<br><br>Did you receive funding for this work?	The author(s) received no specific funding for this work.
**Competing Interests**<br><br>Use the instructions below to enter a competing interest statement for this submission. On behalf of all authors, disclose any competing interests that could be perceived to bias this work—acknowledging all financial support and any other relevant financial or non-financial competing interests.<br><br>This statement is required for submission and **will appear in the published article** if the submission is accepted. Please make sure it is accurate and that any funding	The authors have declared that no competing interests exist

Powered by Editorial Manager® and ProduXion Manager® from Aries Systems Corporation

sources listed in your Funding Information later in the submission form are also declared in your Financial Disclosure statement.<br><br>View published research articles from *PLOS ONE* for specific examples.<br><br>**NO authors have competing interests**<br><br>Enter: *The authors have declared that no competing interests exist.*<br><br>**Authors with competing interests**<br><br>Enter competing interest details beginning with this statement:<br><br>*I have read the journal's policy and the authors of this manuscript have the following competing interests: [insert competing interests here]*<br><br>* typeset	
**Ethics Statement**<br><br>Enter an ethics statement for this submission. This statement is required if the study involved:<br><br>• Human participants<br>• Human specimens or tissue<br>• Vertebrate animals or cephalopods<br>• Vertebrate embryos or tissues<br>• Field research<br><br>Write "N/A" if the submission does not require an ethics statement.<br><br>General guidance is provided below. Consult the submission guidelines for detailed instructions. **Make sure that all information entered here is included in the Methods section of the manuscript.**	N/A

**Format for specific study types**<br><br>**Human Subject Research (involving human participants and/or tissue)**<br>• Give the name of the institutional review board or ethics committee that approved the study<br>• Include the approval number and/or a statement indicating approval of this research<br>• Indicate the form of consent obtained (written/oral) or the reason that consent was not obtained (e.g. the data were analyzed anonymously)<br><br>**Animal Research (involving vertebrate animals, embryos or tissues)**<br>• Provide the name of the Institutional Animal Care and Use Committee (IACUC) or other relevant ethics board that reviewed the study protocol, and indicate whether they approved this research or granted a formal waiver of ethical approval<br>• Include an approval number if one was obtained<br>• If the study involved *non-human primates*, add *additional details* about animal welfare and steps taken to ameliorate suffering<br>• If anesthesia, euthanasia, or any kind of animal sacrifice is part of the study, include briefly which substances and/or methods were applied<br><br>**Field Research**<br><br>Include the following details if this study involves the collection of plant, animal, or other materials from a natural setting:<br>• Field permit number<br>• Name of the institution or relevant body that granted permission	
**Data Availability**<br><br>Authors are required to make all data underlying the findings described fully available, without restriction, and from the time of publication. PLOS allows rare exceptions to address legal and ethical concerns. See the PLOS Data Policy and FAQ for detailed information.	Yes - all data are fully available without restriction

A Data Availability Statement describing where the data can be found is required at submission. Your answers to this question constitute the Data Availability Statement and **will be published in the article**, if accepted.<br><br>**Important:** Stating 'data available on request from the author' is not sufficient. If your data are only available upon request, select 'No' for the first question and explain your exceptional situation in the text box.<br><br>Do the authors confirm that all data underlying the findings described in their manuscript are fully available without restriction?	
**Describe where the data may be found in full sentences. If you are copying our sample text, replace any instances of XXX with the appropriate details.**<br><br>• If the data are **held or will be held in a public repository**, include URLs, accession numbers or DOIs. If this information will only be available after acceptance, indicate this by ticking the box below. For example: *All XXX files are available from the XXX database (accession number(s) XXX, XXX.*).<br>• If the data are all contained **within the manuscript and/or Supporting Information files**, enter the following: *All relevant data are within the manuscript and its Supporting Information files.*<br>• If neither of these applies but you are able to provide **details of access elsewhere**, with or without limitations, please do so. For example:<br><br>*Data cannot be shared publicly because of [XXX]. Data are available from the XXX Institutional Data Access / Ethics Committee (contact via XXX) for researchers who meet the criteria for access to confidential data.*<br><br>*The data underlying the results presented in the study are available from (include the name of the third party*	The data underlying the results presented in the study are available from the corresponding author

and contact information or URL).<br>• This text is appropriate if the data are owned by a third party and authors do not have permission to share the data.<br><br>* typeset	
Additional data availability information:	

Powered by Editorial Manager® and ProduXion Manager® from Aries Systems Corporation

Click here to access/download;Manuscript;Ngompe et al.2.docx

Yield-based drought tolerance indices in cowpea exposed to stress at vegetative and flowering setting stages identifies sensitive growth phase and drought tolerant genotypes in prospect to crop improvement

Toscani Ngompe-Deffo, Eric Bertrand Kouam*, Marie Solange Mamdou, Raba Allah-To Bara, Asafor Henry Chotangui, Adamou Souleymanou, Honore Beyegue-Djonko

Genetics, Biotechnology, Agriculture and Plant Production Research Unit, Department of Crop Science, Faculty of Agronomy and Agricultural Science, University of Dschang, P.O Box 222 Dschang, Cameroon

*****Corresponding author*: ericbkouam@yahoo.com

Abstract

Drought stress following climate change is likely a scenario that will have to face crop growers in tropical regions. In mitigating this constraint, the best option should the selection and use of resilient varieties that can withstand drought threats. Therefore, a pot experiment was conducted under greenhouse conditions at the Research and Teaching Farm of the Faculty of Agronomy and Agricultural Sciences of the University of Dschang, with the objectives of identifying sensitive growth stage and drought-tolerant genotypes following vegetative and early flowering induced stresses, with the help of yield-based selection indices. Eighty-eight cowpea genotypes from the sahelian and western regions of Cameroon were subjected to drought stress at vegetative (VDS) and

flowering setting (FDS) stages by withholding water for 28 days, using a split plot design with two factors and three replications. Seed yields under stress (Ys) and non-stress (Yp) conditions were recorded. Fifteen drought indices were calculated for the two drought stress levels against the yield from non-stress plants. Drought intensity indice (DII) under VDS and FDS were 0.71 and 0.84 respectively, indicating severe drought stress for both stages. However, flowering setting stage was significantly more sensitive to drought stress compared to vegetative stage. Based on PCA and correlation analysis, the indices STI, REI, GMP, MP, YI and HM correlated strongly with yield under stress and non-stress conditions and are therefore suitable to discriminate high-yielding and tolerant genotypes under both stress and non-stress conditions. Either under VDS and FDS, CP-016 exhibited an outstanding performance under drought stress and was revealed as the most drought tolerant genotype as shown by ranking, PCA and cluster analysis. Taking into account all indices, the top five genotypes namely CP 016, CP 021, MTA22, CP 056 and CP060 were identified as the most drought-tolerant genotypes under VDS. For stress activated at flowering setting stage (FDS), CP-016, CP-056, CP-021, CP-028 and MTA-22 were the top five most drought-tolerant genotypes. Several genotypes with valueless Ys and rank among which CP-037, NDT-001, CP036, CP-034, NDT-002, CP-031, NDT-011 were identified as highly drought sensitive with low yield stability. This study identified the most sensitive stage and drought tolerant genotypes that are proposed for genetic improvement of cowpea.

Key worlds: Cowpea genotypes, Drought stress, Drought tolerance, Growth stage, Selection indices

Introduction

Cowpea, of scientific name *Vigna unguiculata* is an important food legume in developing countries, mostly in sub-Saharan Africa, Asia, Central and South America [1, 2]. Cowpea has been referred to as "poor man's meat" because of its high protein content, 20 to 25% [3, 4], as "hungry season crop" because, when grown together with cereals, it is the first crop to be harvested [5]. Owing to its capacity of fixing atmospheric nitrogen, cowpea tolerates low soil fertility and its inclusion in crop rotations makes it possible to meet the nitrogen fertilizer needs of subsequent crops [6]. This characteristic helps increasing the yields of crops and subsequently contributes to the sustainability of cropping systems [7-9]. Cowpea young leaves, pods and peas contain vitamins and minerals useful for human consumption and animal feeding [3, 10]. Sub-Saharan Africa accounts for more than 64% of the world's cowpea production, estimated to about 12.5 million tons [11]. In Cameroon the national production is estimated to about 110,000 tons from a planted area of 105,000 hectares [12]. At the regional level in Cameroon, cowpea is highly produced in the Sahelian region, ordinarily for trade and feeding. The crop there ranks second after groundnuts in the category of leguminous crops [13]. Grain yield in this region ranges from 300 to 500 kg/ha in farmers' fields and 1,200 to 2,000 kg/ha in research stations which use improved varieties [13]. Cowpea is also significantly produced in the Western region mostly for local consumption and cultural considerations [14].

Drought also known as water deficit is one of the abiotic factor limiting significantly crop production [15]. Many authors qualify drought as the most single devastating environmental stress that decrease crop productivity [16, 17]. This moisture stress condition seems to increase in arid and semi-arid regions and is to be highly considered for future climate change scenarios [18, 19]. Cowpea itself

according to the literature can withstand dry environmental conditions at some extent when compared to other crops [20]. This demonstrates its tolerance to water deficit conditions that could have been very disastrous to many other annual crops. When resuming watering after a drought spell, the yield of cowpea according to report of Turk et al. [21] is similar or different to that of a normally irrigated plants and will be depending the growth stage where the plant were exposed to drought. Cowpea is heavily damaged by repeated water deficits [22]. Some researchers like Benjamin et al. [23] and Demirevska et al. [24] pointed that crop failure often occurs because of the unavailability of water at some critical growth stages. In common bean, drought stress during flowering and post-flowering stages result of reductions of 60 to 99% in yield [25]. Early maturing cowpea genotypes are more susceptible to water deficit during flowering and fruit setting phases [26]. Drought reduces cowpea potential from 1000 kg of grains/ha in normal condition, to around 360 kg/ha when stress sets-in during pre-flowering [27]. This drastic reduction is likely to be accounted to the unavailability of high yielding varieties resistant to moisture stress [28, 29]. Varieties differentiation is observed in yield as well as in response to drought stress in many crops. Pejic et al. [22] revealed significant difference between cowpea varieties when exposed to water deficit conditions.

In order to ensure future food needs for the increasing world population, the approach involving a better use of available water for the development of crop is to be highly considered. With climate change making the earth more warn, using varieties which needs less amount of water and more tolerance to drought will be the best option for crop production ~~to feeding people~~ [30]. Yield loss is the main concern of plant breeders as farmers usually experience low yield performance under stress conditions. Therefore, drought indices which provide a measure of drought based on loss of yield under drought-conditions in comparison to normal conditions have been largely used for screening drought tolerant genotypes in many crops including cowpea [22], common bean [31-33], maize [34,

35], wheat [36] and sorghum [37]. According to Fernandez [38], the best indices are those having high correlation with grain yield in both stress and non-stress conditions and the best selection in drought condition could be to isolate genotypes having desirable and similar yield in stress and non-tress condition. Although, there are several reports on the association of the indices with drought tolerance in many crops, very few studies were carried out on cowpea and using limited number of genotypes. The evaluation of a large quantity of genotypes from various sources is the novelty of this study. This is particularly important for exploiting the available genetic variation and identifying the most tolerant genotypes that will be valued by cowpea breeders in developing superior cultivars. The objectives of this study were to assess eighty-eight cowpea genotypes under non stress and stress conditions activated at the vegetative and flowering setting stages in order to: (i) identify the sensitive growth stage in cowpea to drought stress by the help of selection indices, (ii) identify high-yielding drought tolerant genotypes using drought tolerance indices.

Materials and Methods

Study site.

The experiment was carried out in the genetic experimental greenhouse at the Research and Teaching Farm of the Faculty of Agronomy and Agricultural Sciences of the University of Dschang, located in the Western Region of Cameroon at 5°20’ North latitude and 10°05’ East longitude, and 1407 m asl. Others characteristics of the study site include an average annual temperature of 21.50 °C and the relative humidity of 76.8%.

Plant material and different water regimes

Eighty eight cowpea accessions used for the study were collected from the Sahelian and the Western highland region of Cameroon. Accessions were from the Institute of Agricultural Research for Development (9 accessions)) and 79 landraces sourced from market places and farmers' fields. Three treatments namely, non-drought stress (NDS), drought stress at vegetative stage (VDS), and drought stress at flowering setting stages (FDS) were applied. Drought stress consisted at withholding water for 28 days at each of the corresponding stage.

Experimental design and procedures

The experimental design was a split plot involving two factors with three replications. The main factor was the water regime with 3 levels: no stress = regular irrigation (NDS), stress at the vegetative stage (VDS, started at around three weeks from planting, when the first trifoliate leaf appeared open and flat on 50% of plants) and stress initiated and flowering setting stage (FDS, when the first open flower appeared on 50% of plants). The subplot consisted of genotypes, 88 in total and constituted the second factor. Plastic pots of 5 litres capacity and perforated at the bottom were filled with top soil collected up to 30 cm depth around the experimental site. Five seeds of each cowpea genotype were planted in each pot, and two weeks after sowing, the seedlings were thinned to three per pot and to two one weeks later. All pots were well watered with two-day interval frequency and up to the initiation of drought stress. One day before starting treatments, soil moisture in each pot was maintained to field capacity for the purpose of keeping uniform soil moisture at each pot. This generated 264 interactions repeated 3 times, giving 792 experimental units. The units were spaced 0.3 m apart within rows and 0.5 m between rows. Replications were separated from each other by 0.5 m. For stressed plants, the protocol of Fernandez [38] was followed with the irrigation completely cut off for 28 days before resuming to normal irrigation. Under non-drought stress condition, plants were regularly watered up to field capacity until physiological maturity..

Drought tolerance indices calculation and data analysis

At harvest, seed were collected on each plant. Weighing followed using pionner sensitive digital balance and seed yield estimated. In addition to the direct measurement, some derived variables that are drought tolerance indices were calculated from primary data for the two drought stress stages from grain yield under the drought-stressed (VDS and (FDS), and non-stressed (NDS) conditions according to various formula that are indicated in Table 1. Seed yield data were recorded for each genotype at both stress and non-stress environment and were subjected to the estimation of the different drought selection indices following appropriate formula and using Microsoft Excel software. Pearson correlation analysis was performed using XLSTAT software computer programs to determine the association between drought tolerance indices and grain yield in both stressed and non-stressed conditions. To determine the most desirable drought-tolerant genotype, rank mean value was given based on each selection indices rank value. This rank mean was calculated by averaging for all selection indices value for the considered genotype. The standard deviation (the deviation of each rank from the mean of rank) of rank mean was also calculated. The rank sum was calculated according to Yahaya et al. [37] using this relationship formula: $Rank\ Sum\ (RS) = Rank\ Mean\ (\bar{R}) + Standard\ Deviation\ of\ Rank (SDR)$. Genotypes with low rank mean and low standard deviation of rank were considered as best drought-tolerant. Besides ranking methods, Principal component and hierarchical clustering analysis from the XLSTAT software were used for the identification of the suitable tolerant genotypes following Fernandez [38] recommendations

Table 1: Code, name and formula applied to estimate the fourteen drought tolerance indices from grain yield under the drought-stressed (Ys) and non-stressed conditions (Yp).

No	Index code	Index name	Formula	Remark	Reference
1	SSI	Stress Susceptibility Index	$SSI = \frac{1-(\frac{Ysi}{Ypi})}{1-(\frac{Ys}{Yp})}$	The greater the index, the greater susceptibility of the genotype to stress.	[39]
2	RDI	Relative Drought Index	$RDI = \frac{\frac{Ysi}{Ypi}}{\frac{Ys}{Yp}}$	the genotype is relatively drought tolerant if RDI > 1 and drought susceptible if RDI < 1	[40]
3	SSPI	Stress Susceptibility Percentage Index	$SSPI = \frac{Ypi - Ysi}{2(Yp)}$	The smaller SSPI, the more relative tolerance	[39]
4	SDI	Sensitivity Drought Index	$SDI = \frac{\text{Ypi} - \text{Ysi}}{\text{Ypi}}$	The genotypes with small value of this index will be more desirable	[38]
5	TOL	Tolerance index	$TOL = \text{Ypi} - \text{Ysi}$	The genotypes with small value of this index will be more desirable	[38]
6	STI	Stress Tolerance Index	$STI = \frac{\text{Ypi} * \text{Ysi}}{(\text{Yp})^2}$	The genotypes with high STI values are more tolerant to drought stress	[38]
7	REI	Relative Efficiency Index	$REI = \frac{Ysi}{Ys} * \frac{Ypi}{Yp}$	The genotypes with high value of this index will be more desirable	[41]
8	GMP	Geometric Mean Productivity	$\text{GMP} = \sqrt{Ypi * Ysi}$	The higher GMP value, the greater the degree of relative tolerance	[38]
9	MP	Mean Productivity	$MP = \frac{\text{Ypi} + \text{Ysi}}{2}$	The genotypes with high value of this index will be more desirable	[38]
10	YSI	Yield Stability Index	$YSI = \frac{\text{Ysi}}{\text{Ypi}}$	The genotypes with high values can be regarded as stable genotypes under stress and non-stress conditions	[42]
11	YI	Yield Index	$YI = \frac{\text{Ysi}}{\text{Ys}}$	The genotypes with high value of this index will be suitable for drought stress condition	[43]
12	PYR	Percentage of Yield Reduction	$PYR = \left(\frac{\text{Ypi} - \text{Ysi}}{\text{Ypi}}\right) * 100$	The genotypes with small value of this index will be more desirable	[44]
13	DTI	Drought Tolerance Index	$DTI = \frac{Ysi(\frac{Ysi}{Ypi})}{Ys}$	genotypes with the highest DTI will be desirable to moisture stress	[45]
14	HM	Harmonic Mean	$HM = \frac{2(Yp * Ys)}{Yp + Ys}$	The genotypes with high value of this index will be more desirable	[46]
15	DII	Drought Intensity Index	$DII = 1 - \frac{\text{Ys}}{\text{Yp}}$	The genotypes with low value of DII will be more desirable under drought conditions	[47]

Results

Drought stress and sensitive growth stage

Compared to controls plants (NDS), plants exposed to VDS and FDS ~~each~~ gave a seed yield significantly lower with differential responses among genotypes. Between VDS and FDS, statistical comparisons of the different indices were performed and are presented in Table 2. Drought intensity index (DII) for VDS was 0.71 and significantly lower compared to 0.84, for FDS. Also, FDS registered significantly higher values for Stress susceptibility percentage index (SSPI=42.59%) and Percentage of yield reduction (PYR=83.64%) when compared to VDS, 36.42% and 70.52% respectively (Table 2). The FDS had significantly low values of Yield under stress (Ys=1.46), Stress tolerance index (STI=0.18), Geometric mean productivity (GMP=2.77), Mean productivity (MP=5.68) and Drought tolerance index (DTI=0.41) when compared to VDS with Ys=2.69, STI=0.33, GMP=4.32, MP=6.29 and DTI=0.51 (Table 2). These results demonstrate that stress induced at the flowering setting stage is more sensitive and impacted negatively cowpea's productivity compared to the stress that is activated during vegetative stage

Table 2: Mean yield under stress and drought tolerance indices for vegetative drought stress (VDS) and flowering setting drought stress (FDS) for cowpea genotypes

	YS	SSI	RDI	SSPI	SDI	TOL	STI	REI	GMP	MP	YSI	YI	PYR	DTI	HM	DII
**VDS**	2.687^{a}	0.926^{a}	1.095^{a}	36.424^{b}	0.706^{b}	7.210^{b}	0.330^{a}	1.226^{a}	4.324^{a}	6.292^{a}	0.295^{a}	1.009^{a}	70.519^{b}	0.513^{a}	3.661^{a}	0.705^{b}
**FDS**	1.466^{b}	0.963^{a}	1.149^{a}	42.593^{a}	0.835^{a}	8.431^{a}	0.176^{b}	1.235^{a}	2.773^{b}	5.681^{b}	0.164^{b}	1.040^{a}	83.635^{a}	0.411^{b}	2.160^{b}	0.836^{a}

YS= Mean value of grain yield under drought stress condition, SSI= Stress susceptibility index, RDI=relative drought index, SSPI= Stress susceptibility percentage index, SDI= Sensitivity drought index, TOL= stress tolerance, STI= Stress tolerance index, REI= The relative efficiency index, GMP= Geometric mean productivity, MP= Mean productivity, YSI= Yield stability index, YI= Yield index, PYR= percentage of yield reduction, DTI= Drought tolerance index, HM= Harmonic mean, DII = Drought Intensity Index, VDS= Vegetative drought stress, FDS= Flowering setting drought stress. In the same column, means followed by the same letter are not significantly different at 0.050 probability level

Drought tolerance indices and ranking of cowpea genotypes

Under VDS and FDS, genotypes (C-9, CP-016, C-10, C-7, CP-018) and (CP-016, CP-042, CP-056) respectively, gave the highest STI, GMP, MP and REI values indicating that these genotypes are high yielding under both drought stress and non-stress conditions. The lowest STI, GMP, MP and REI values were obtained by NDT-022 and NDT-012 under drought stress at both stages, signifying their sensitivity to drought. CP-016 and C-9 also displayed highest YI, HM, and grain yield under the two stress conditions. However, NDT-022 and NDT-012 showed the lowest estimate for these indices during the VDS (Table 3). With regards to FDS, genotypes CP-016 and CP-056 displayed highest YI, HM, and grain yield while NDT-022 and NDT-012 showed the lowest values (Table 4). Based on YSI and RDI indices, genotypes CP-046, MTA-22 and CP-060, were found with highest values while many genotypes including CP-037displayed the lowest amount for this indices during VDS (Table 3). The SSPI and TOL indices for VDS were low for CP-046, MTA-22 and CP-060 and high for C-9. These indices were low for NDT-011, CP-049 and CP-025 and high for E, CP-004 and C-7 during FDS (Table 3 and 4). Also, CP-046, MTA-22 and CP-060 had low values, CP-043 and CP-016 had high values for PYR, SSI and SDI during VDS and at FDS, lowest values for PYR, SSI and SDI were for CP-056, CP-016 and CP-049 and the high values for many genotypes including CP-043, CP-006, C and D (Tables 3 and 4). Drought tolerance index (DTI) at VDS had high values for CP-016, MTA-22, CP-046 and CP-060 and the lowest amount were for many genotypes including CP-037 and CP-043 while at the FDS, DTI were high for CP-016, CP-056, CP-021 and CP-049 and the lowest values recorded for CP-037 and CP-043, CP-006 (Tables 3 and 4). Taking into consideration all indices, the top five genotypes namely CP 016, CP 021, MTA22, CP 056 and CP060 exhibited the best sum of ranks and hence, they were identified as the most drought-tolerant genotypes for drought stress

induced at the vegetative stage (VDS, Table 3). For stress activated at flowering setting stage (FDS), the top five most drought-tolerant genotypes were CP-016, CP-056, CP-021, CP-028 and MTA-22 (Table 4). However, genotypes namely CP-037, NDT-011, CP-036, NDT-001 and NDT-002 were the top five most drought susceptible plants at the vegetative stage (Table 3) while the top five susceptible genotypes at the flowering setting stage were CP-037, CP-034, NDT-001, CP-036 and CP-031 (Table 4)

Table 3: Mean grain yield (g/plant) under VDS (Ysi_1) and NDS (Ypi) conditions as well as measures of different drought tolerance indices, rank mean, SD of rand mean and rank sum for 88 cowpea genotypes.

Genotype	Ypi	Ysi_1	SSI	RDI	SSPI	SDI	TOL	STI	REI	GMP	MP
CP-070	9.72(35)	3.19(33)	0.88(38)	1.22(39)	32.98(52)	0.67(39)	6.53(51)	0.316(29)	1.18(29)	5.57(29)	6.45(35)
CP-071	8.992(42)	1.35(55)	1.11(52)	0.56(52)	38.63(55)	0.85(52)	7.65(54)	0.123(50)	0.45(50)	3.47(50)	5.17(53)
CP-044	10.98(28)	3.78(28)	0.86(35)	1.28(36)	36.41(54)	0.66(36)	7.21(53)	0.423(23)	1.57(23)	6.44(23)	7.38(29)
CP-042	19.76(4)	3.22(32)	1.09(50)	0.61(50)	83.57(83)	0.84(50)	16.54(81)	0.649(14)	2.41(14)	7.97(14)	11.49(6)
CP-018	15.20(13)	5.70(11)	0.81(30)	1.39(31)	47.99(68)	0.62(31)	9.49(66)	0.884(6)	3.28(6)	9.30(6)	10.45(9)
CP-043	10.50(30)	0.00(69)	1.30(69)	0.00(69)	53.03(71)	1.00(69)	10.49(69)	0(69)	0(69)	0(69)	5.25(52)
CP-056	10.19(32)	6.70(9)	0.45(11)	2.44(12)	17.62(25)	0.34(12)	3.48(25)	0.697(12)	2.59(12)	8.26(12)	8.45(17)
CP-067	11.74(24)	0.62(63)	1.23(65)	0.19(65)	56.22(73)	0.95(65)	11.13(71)	0.073(56)	0.27(56)	2.68(56)	6.18(36)
CP-002	6.24(65)	3.78(27)	0.51(14)	2.25(15)	12.41(16)	0.39(15)	2.45(16)	0.24(37)	0.89(37)	4.85(37)	5.012(56
CP-006	8.48(45)	2.94(36)	0.85(34)	1.29(35)	27.97(40)	0.65(35)	5.53(40)	0.25(35)	0.94(35)	4.99(35)	5.71(44)
CP-037	1.92(88)	0.00(69)	1.30(69)	0.00(69)	9.70(11)	1.00(69)	1.92(11)	0.00(69)	0(69)	0(69)	0.96(88)
CP-035	6.24(66)	1.63(49)	0.96(45)	0.97(46)	23.28(32)	0.74(46)	4.61(32)	0.104(54)	0.38(54)	3.19(54)	3.93(65)
CP-058	9.44(38)	4.55(19)	0.67(19)	1.79(20)	24.70(36)	0.52(20)	4.88(36)	0.438(22)	1.63(22)	6.55(22)	6.99(30)
CP-034	19.68(5)	2.19(43)	1.16(58)	0.41(58)	88.35(85)	0.89(58)	17.48(83)	0.440(21)	1.63(21)	6.56(21)	10.94(8)
CP-009	14.77(17)	5.32(13)	0.83(32)	1.34(33)	47.69(66)	0.64(33)	9.44(64)	0.803(7)	2.98(7)	8.87(7)	10.04(10
CP-001	9.36(39)	3.88(24)	0.76(28)	1.54(29)	27.71(39)	0.59(29)	5.48(39)	0.370(26)	1.37(26)	6.02(26)	6.62(33)
CP-017	11.34(26)	3.51(30)	0.90(41)	1.15(42)	39.53(56)	0.69(42)	7.82(55)	0.406(24)	1.51(24)	6.30(24)	7.42(28)
CP-019	2.74(84)	1.65(48)	0.52(15)	2.23(16)	5.53(6)	0.40(16)	1.10(6)	0.046(62)	0.17(62)	2.12(62)	2.19(82)
CP-033	8.93(43)	6.62(10)	0.34(7)	2.75(7)	11.68(15)	0.26(7)	2.31(15)	0.603(16)	2.24(16)	7.68(16)	7.77(25)
CP-045	8.08(53)	2.60(40)	0.88(39)	1.20(40)	27.69(38)	0.68(40)	5.48(38)	0.214(39)	0.80(39)	4.58(39)	5.34(50)
CP-060	8.32(48)	7.59(5)	0.11(3)	3.39(3)	3.68(3)	0.09(3)	0.72(3)	0.644(15)	2.39(15)	7.94(15)	7.96(23)
CP-014	14.34(19)	7.76(3)	0.60(17)	2.01(18)	33.22(53)	0.46(18)	6.57(52)	1.135(4)	4.22(4)	10.54(4)	11.04(7)
CP-041	5.59(74)	3.82(26)	0.41(10)	2.54(11)	8.93(10)	0.32(11)	1.76(10)	0.218(38)	0.81(38)	4.62(38)	4.71(59)
CP-057	11.20(27)	4.84(18)	0.74(23)	1.61(24)	32.13(50)	0.57(24)	6.36(49)	0.553(19)	2.06(19)	7.36(19)	8.02(22)
CP-007	8.28(50)	2.05(45)	0.98(46)	0.92(47)	31.49(46)	0.75(47)	6.23(46)	0.173(43)	0.64(43)	4.11(43)	5.16(54)
CP-030	6.88(57)	0.91(58)	1.13(54)	0.49(54)	30.15(44)	0.87(54)	5.96(44)	0.064(58)	0.25(58)	2.50(58)	3.89(66)

CP-020	15.44(12)	0.82(60)	1.23(64)	0.20(64)	73.88(78)	0.95(64)	14.62(76)	0.128(49)	0.47(49)	3.54(49)	8.13(20)
CP-013	6.04(70)	2.78(37)	0.70(20)	1.71(21)	16.49(24)	0.54(21)	3.26(24)	0.171(44)	0.63(44)	4.09(44)	4.40(63)
CP-012	14.56(18)	5.21(15)	0.84(33)	1.33(34)	47.23(63)	0.64(34)	9.34(62)	0.774(8)	2.87(8)	8.71(8)	9.88(12)
CP-046	7.80(55)	7.42(6)	0.06(1)	3.53(1)	1.92(1)	0.05(1)	0.38(1)	0.591(17)	2.19(17)	7.61(17)	7.62(26)
CP-003	9.58(37)	0.35(65)	1.26(66)	0.14(66)	46.62(61)	0.96(66)	9.22(60)	0.034(63)	0.12(63)	1.83(63)	4.97(57)
CP-005	4.57(77)	1.38(54)	0.91(43)	1.12(44)	16.13(23)	0.70(44)	3.19(23)	0.064(57)	0.26(57)	2.50(57)	2.972(76
CP-004	25.04(2)	2.98(35)	1.15(56)	0.44(56)	111.43(87)	0.88(56)	22.05(85)	0.762(11)	2.83(11)	8.6411)	14.01(3)
CP-054	11.52(25)	0.25(67)	1.28(67)	0.08(67)	56.95(74)	0.98(67)	11.27(72)	0.029(65)	0.11(65)	1.69(65)	5.88(42)
CP-021	10.35(31)	7.24(7)	0.39(9)	2.60(9)	15.72(22)	0.30(9)	3.11(22)	0.765(9)	2.84(9)	8.66(9)	8.79(15)
CP-027	16.22(10)	1.10(57)	1.21(60)	0.25(60)	76.40(81)	0.93(60)	15.12(79)	0.182(41)	0.68(41)	4.22(41)	8.66(16)
MTA-22	8.48(46)	7.76(4)	0.11(2)	3.40(2)	3.64(2)	0.08(2)	0.72(2)	0.671(13)	2.49(13)	8.11(13)	8.12(21)
CP-036	16.62(8)	0.00(69)	1.30(69)	0.00(69)	83.99(84)	1.00(69)	16.62(82)	0(69)	0(69)	0(69)	8.34(18)
CP-026	16.72(7)	1.93(46)	1.15(57)	0.43(57)	74.74(79)	0.88(57)	14.79(77)	0.328(27)	1.22(27)	5.67(27)	9.32(13)
CP-025	2.21(86)	0.70(61)	0.89(40)	1.18(41)	7.62(9)	0.68(41)	1.51(9)	0.015(67)	0.06(67)	1.24(67)	1.45(85)
CP-069	8.38(47)	2.50(41)	0.92(44)	1.11(45)	29.75(43)	0.70(45)	5.88(43)	0.213(40)	0.79(40)	4.57(40)	5.44(49)
CP-029	8.24(51)	3.00(34)	0.83(31)	1.35(32)	26.47(37)	0.64(32)	5.24(37)	0.252(36)	0.93(36)	4.97(36)	5.62(47)
CP-049	5.91(71)	0.35(66)	1.23(62)	0.22(62)	28.09(41)	0.94(62)	5.56(41)	0.021(66)	0.08(66)	1.44(66)	3.132(73
CP-008	6.40(62)	0.00(69)	1.30(69)	0.00(69)	32.33(51)	1.00(69)	6.4(50)	0(69)	0(69)	0(69)	3.20(70)
CP-048	6.51(61)	4.04(23)	0.49(13)	2.30(14)	12.49(17)	0.38(14)	2.47(17)	0.268(34)	0.99(34)	5.13(34)	5.28(51)
CP-068	6.72(59)	5.57(12)	0.22(5)	3.08(5)	5.82(7)	0.17(5)	1.15(7)	0.382(25)	1.42(25)	6.11(25)	6.14(38)
CP-016	18.72(6)	15.05(1)	0.26(6)	2.99(6)	18.55(28)	0.20(6)	3.67(28)	2.876(2)	10.68(2)	16.78(2)	16.88(2)
CP-031	3.26(82)	2.32(42)	0.38(8)	2.64(8)	4.77(5)	0.29(8)	0.94(5)	0.077(55)	0.28(55)	2.75(55)	2.79(78)
CP-028	8.30(49)	3.60(29)	0.74(22)	1.61(23)	23.78(33)	0.57(23)	4.70(33)	0.305(30)	1.13(30)	5.46(30)	5.95(40)
CP-024	8.24(52)	3.49(31)	0.75(25)	1.57(26)	24.01(34)	0.58(26)	4.75(34)	0.293(33)	1.08(33)	5.36(33)	5.86(43)
C	4.00(78)	2.77(38)	1.05(48)	2.57(10)	6.22(8)	0.31(10)	1.23(8)	0.113(51)	0.42(51)	3.32(51)	3.38(69)
D	6.07(69)	5.22(14)	0.18(4)	3.20(4)	4.28(4)	0.14(4)	0.85(4)	0.323(28)	1.21(28)	5.63(28)	5.65(46)
NDT-003	3.81(79)	1.61(50)	0.75(26)	1.57(27)	11.11(14)	0.58(27)	2.2(14)	0.062(60)	0.23(60)	2.47(60)	2.71(79)
F	6.22(68)	0.00(69)	1.30(69)	0.00(69)	31.45(45)	1.00(69)	6.22(45)	0(69)	0(69)	0(69)	3.11(75)
B	6.24(67)	0.00(69)	1.30(69)	0.00(69)	31.53(47)	1.00(69)	6.24(47)	0(69)	0(69)	0(69)	3.12(74)
NDT-010	14.98(14)	5.00(17)	0.87(37)	1.24(38)	50.40(70)	0.67(38)	9.97(68)	0.764(10)	2.84(10)	8.65(10)	9.98(11)
NDT-016	3.62(80)	1.50(52)	0.76(27)	1.54(28)	10.69(13)	0.59(28)	2.11(13)	0.055(61)	0.21(61)	2.32(61)	2.56(80)
NDT-045	6.80(58)	4.29(21)	0.48(12)	2.34(13)	12.69(18)	0.37(13)	2.51(18)	0.297(31)	1.11(31)	5.39(31)	5.54(48)
NDT-004	10.90(29)	1.42(53)	1.13(55)	0.48(55)	47.90(67)	0.87(55)	9.48(65)	0.157(46)	0.58(46)	3.92(46)	6.16(37)
NDT-002	14.88(16)	0.00(69)	1.30(69)	0.00(69)	75.18(80)	1.00(69)	14.88(78)	0(69)	0(69)	0(69)	7.44(27)
E	14.98(15)	0.86(59)	1.23(63)	0.21(63)	71.30(77)	0.94(63)	14.11(75)	0.132(48)	0.49(48)	3.597(48)	7.92(24)
C-9	42.63(1)	8.40(2)	1.05(47)	0.73(48)	172.95(88)	0.80(48)	34.23(86)	3.656(1)	13.59(1)	18.92(1)	25.52(1)
C-7	23.38(3)	4.47(20)	1.05(49)	0.71(49)	95.51(86)	0.81(49)	18.90(84)	1.067(5)	3.96(5)	10.22(5)	13.93(4)
NDT-029	3.20(83)	0.44(64)	1.12(53)	0.51(53)	13.94(20)	0.86(53)	2.76(20)	0.014(68)	0.05(68)	1.18(68)	1.82(83)
C-14	10.18(33)	1.60(51)	1.10(51)	0.58(51)	43.37(58)	0.84(51)	8.58(57)	0.166(45)	0.62(45)	4.03(45)	5.89(41)
C-10	16.36(9)	7.00(8)	0.75(24)	1.59(25)	47.2(64)	0.57(25)	9.36(63)	1.169(3)	4.34(3)	10.70(3)	11.68(5)
C-18	13.52(20)	4.17(22)	0.90(42)	1.15(43)	47.22(62)	0.69(43)	9.34(61)	0.576(18)	2.14(18)	7.51(18)	8.84(14)
C-19	9.60(36)	0.64(62)	1.22(61)	0.25(61)	45.27(59)	0.93(61)	8.96(58)	0.062(59)	0.24(59)	2.47(59)	5.12(55)
NDT-015	13.28(21)	0.00(69)	1.30(69)	0.00(69)	67.09(76)	1.00(69)	13.28(74)	0(69)	0(69)	0(69)	6.64(32)

NDP-017	9.36(40)	0.00(69)	1.30(69)	0.00(69)	47.29(65)	1.00(69)	9.36(63)	0(69)	0(69)	0(69)	4.68(60)
NDT-005	9.92(34)	0.00(69)	1.30(69)	0.00(69)	50.12(69)	1.00(69)	9.92(67)	0(69)	0(69)	0(69)	4.96(58)
NDT-006	5.61(73)	1.90(47)	0.86(36)	1.26(37)	18.73(29)	0.66(37)	3.71(29)	0.108(53)	0.40(53)	3.26(53)	3.75(67)
NDT-025	8.80(44)	5.04(16)	0.56(16)	2.13(17)	19.00(30)	0.43(17)	3.76(30)	0.452(20)	1.68(20)	6.65(20)	6.92(31)
NDT-001	2.56(85)	0.00(69)	1.30(69)	0.00(69)	12.93(19)	1.00(69)	2.56(19)	0(69)	0(69)	0(69)	1.28(86)
A	7.50(56)	3.84(25)	0.64(18)	1.90(19)	18.51(27)	0.49(19)	3.66(27)	0.295(32)	1.09(32)	5.37(32)	5.67(45)
NDT-009	12.21(22)	0.00(69)	1.30(69)	0.00(69)	61.68(75)	1.00(69)	12.21(73)	0(69)	0(69)	0(69)	6.10(39)
NDT-027	4.96(75)	2.19(44)	0.73(21)	1.64(22)	13.98(21)	0.56(22)	2.76(21)	0.111(52)	0.41(52)	3.29(52)	3.57(68)
NDT-011	2.08(87)	0.00(69)	1.30(69)	0.00(69)	10.51(12)	1.00(69)	2.08(12)	0(69)	0(69)	0(69)	1.04(87)
NDT-008	11.98(23)	1.20(56)	1.17(59)	0.37(59)	54.48(72)	0.90(59)	10.78(70)	0.146(47)	0.54(47)	3.79(47)	6.59(34)
C-8	6.59(60)	2.67(39)	0.77(29)	1.51(30)	19.80(31)	0.59(30)	3.92(31)	0.179(42)	0.67(42)	4.19(42)	4.63(61)
NDT-013	16.16(11)	0.19(68)	1.29(68)	0.04(68)	80.67(82)	0.99(68)	15.96(80)	0.031(64)	0.12(64)	1.76(64)	8.17(19)
NDT-022	3.54(81)	0.00(69)	1.30(69)	0.00(69)	17.86(26)	1.00(69)	3.53(26)	0(69)	0(69)	0(69)	1.76(84)
NDT-018	4.80(76)	0.00(69)	1.30(69)	0.00(69)	24.25(35)	1.00(69)	4.8(35)	0(69)	0(69)	0(69)	2.40(81)
NDT-026	9.10(41)	0.00(69)	1.30(69)	0.00(69)	46.00(60)	1.00(69)	9.1&(59)	0(69)	0(69)	0(69)	4.55(62)
NDT-020	6.32(63)	0.00(69)	1.30(69)	0.00(69)	31.93(48)	1.00(69)	6.32(48)	0(69)	0(69)	0(69)	3.16(71)
NDT-023	7.89(54)	0.00(69)	1.30(69)	0.00(69)	39.85(57)	1.00(69)	7.88(56)	0(69)	0(69)	0(69)	3.94(64)
NDT-012	5.79(72)	0.00(69)	1.30(69)	0.00(69)	29.26(42)	1.00(69)	5.79(42)	0(69)	0(69)	0(69)	2.89(77)
NDT-025 B	6.32(64)	0.00(69)	1.30(69)	0.00(69)	31.93(49)	1.00(69)	6.32(48)	0(69)	0(69)	0(69)	3.16(72)

Values in the parentheses refer to the ranks of the genotype for each index, YSi1= Mean value of grain yield under drought stress condition, Ypi=mean value of yield under non-stress condition, SSI= Stress susceptibility index, RDI=relative drought index, SSPI= Stress susceptibility percentage index, SDI= Sensitivity drought index, TOL= stress tolerance, STI= Stress tolerance index, REI= The relative efficiency index, GMP= Geometric mean productivity, MP= Mean productivity, YSI= Yield stability index, YI= Yield index, PYR= percentage of yield reduction, DTI= Drought tolerance index, HM= Harmonic mean, DII = Drought Intensity Index

Table 4: Mean grain yield (g/plant) under FDS (Ysi_2) and NDS (Ypi) conditions as well as measures of different drought tolerance indices, rank mean, SD of rand mean and rank sum for 88 cowpea genotypes.

Accessions	Ypi	Ysi_2	SSI	RDI	SSPI	SDI	TOL	STI	REI	GMP	MP
CP-070	9.72(35)	3.27(13)	0.992(40)	5.622(1)	32.59(40)	0.663(15)	6.45(40)	0.32(13)	2.27(13)	5.63(13)	6.49(28)
CP-071	8.992(42)	2.2(21)	0.989(38)	5.543(2)	34.31(43)	0.755(25)	6.79(42)	0.201(26)	1.41(26)	4.44(26)	5.59(36)
CP-044	10.98(28)	2.2(22)	0.913(29)	5.179(3)	44.37(53)	0.799(32)	8.78(52)	0.246(21)	1.72(21)	4.91(21)	6.59(27)
CP-042	19.76(4)	5.58(4)	0.819(20)	4.117(4)	71.61(77)	0.717(22)	14.17(76)	1.12(2)	7.91(2)	10.50(2)	12.67(4)
CP-018	15.20(13)	2.8(18)	0.931(32)	4.027(5)	62.64(72)	0.815(35)	12.40(71)	0.43(10)	3.05(9)	6.52(10)	9.00(13)
CP-043	10.50(30)	0(53)	1.14(53)	3.654(6)	53.02(65)	1(53)	10.49(64)	0(53)	0(53)	0(53)	5.24(38)
CP-056	10.19(32)	8.16(2)	0.22(1)	3.252(7)	10.26(8)	0.199(1)	2.03(8)	0.84(3)	5.96(3)	9.11(3)	9.17(10)
CP-067	11.74(24)	0.704(46)	1.074(49)	3.138(8)	55.77(67)	0.940(49)	11.04(66)	0.084(39)	0.58(39)	2.87(39)	6.22(29)
CP-002	6.24(65)	1.912(27)	0.791(15)	3.101(9)	21.86(21)	0.693(17)	4.31(21)	0.121(33)	0.85(33)	3.45(33)	4.07(61)
CP-006	8.48(45)	0(53)	1.14(53)	2.879(10)	42.84(52)	1(53)	8.48(51)	0(53)	0(53)	0(53)	4.24(57)
CP-037	1.92(88)	0(53)	1.14(53)	2.861(11)	9.70(6)	1(53)	1.92(6)	0(53)	0(53)	0(53)	0.96(88)
CP-035	6.24(66)	0(53)	1.14(53)	2.740(12)	31.52(35)	1(53)	6.24(35)	0(53)	0(53)	0(53)	3.15(71)

CP-058	9.44(38)	2.448(20)	0.845(22)	2.586(13)	35.32(44)	0.740(24)	6.99(43)	0.235(23)	1.65(23)	4.80(23)	5.94(32)
CP-034	19.68(5)	0(53)	1.14(53)	2.584(14)	99.42(85)	1(53)	19.68(84)	0(53)	0(53)	0(53)	9.84(6)
CP-009	14.77(17)	1.976(24)	0.988(37)	2.362(15)	64.62(73)	0.86(39)	12.79(72)	0.29(14)	2.09(14)	5.40(14)	8.37(15)
CP-001	9.36(39)	0(53)	1.14(53)	2.318(16)	47.28(58)	1(53)	9.36(57)	0(53)	0(53)	0(53)	4.68(49)
CP-017	11.34(26)	0(53)	1.14(53)	2.151(17)	57.27(68)	1(53)	11.33(67)	0(53)	0(53)	0(53)	5.66(34)
CP-019	2.74(84)	0.462(52)	0.949(34)	2.092(18)	11.52(9)	0.831(37)	2.28(9)	0.012(52)	0.09(52)	1.12(52)	1.60(84)
CP-033	8.93(43)	0(53)	1.14(53)	2.086(19)	45.10(55)	1(53)	8.92(54)	0(53)	0(53)	0(53)	4.46(52)
CP-045	8.08(53)	0(53)	1.14(53)	2.048(20)	40.82(49)	1(53)	8.08(48)	0(53)	0(53)	0(53)	4.04(62)
CP-060	8.32(48)	1.92(26)	0.878(26)	1.999(21)	32.33(39)	0.769(29)	6.40(39)	0.163(31)	1.14(31)	3.99(31)	5.12(40)
CP-014	14.34(19)	3.36(10)	0.874(24)	1.985(22)	55.45(66)	0.765(27)	10.97(65)	0.49(9)	3.45(8)	6.94(9)	8.84(14)
CP-041	5.59(74)	1.088(40)	0.92(30)	1.975(23)	22.75(23)	0.805(33)	4.50(23)	0.062(44)	0.42(44)	2.46(44)	3.34(67)
CP-057	11.20(27)	3.337(11)	0.801(16)	1.821(24)	39.72(47)	0.702(18)	7.86(46)	0.38(12)	2.68(11)	6.11(12)	7.26(23)
CP-007	8.28(50)	1.958(25)	0.871(23)	1.718(25)	31.94(38)	0.763(26)	6.33(38)	0.165(30)	1.16(30)	4.02(30)	5.11(41)
CP-030	6.88(57)	1.328(37)	0.921(31)	1.661(26)	28.04(28)	0.807(34)	5.55(28)	0.093(36)	0.65(36)	3.02(36)	4.11(59)
CP-020	15.44(12)	3.504(6)	0.882(27)	1.646(27)	60.30(70)	0.773(30)	11.93(69)	0.54(7)	3.87(6)	7.35(7)	9.47(8)
CP-013	6.04(70)	1.72(32)	0.816(19)	1.641(28)	21.82(19)	0.715(21)	4.33(19)	0.106(35)	0.74(35)	3.22(35)	3.88(65)
CP-012	14.56(18)	0(53)	1.14(53)	1.621(29)	73.56(78)	1(53)	14.56(77)	0(53)	0(53)	0(53)	7.28(22)
CP-046	7.80(55)	2.872(17)	0.721(13)	1.593(30)	24.92(26)	0.632(14)	4.93(26)	0.228(24)	1.60(24)	4.73(24)	5.33(37)
CP-003	9.58(37)	0.64(49)	1.065(48)	1.457(31)	45.16(56)	0.933(48)	8.93(55)	0.063(43)	0.43(43)	2.47(43)	5.10(42)
CP-005	4.57(77)	0(53)	1.14(53)	1.406(32)	23.07(24)	1(53)	4.56(24)	0(53)	0(53)	0(53)	2.28(79)
CP-004	25.04(2)	0.712(45)	1.109(52)	1.366(34)	122.91(87)	0.971(52)	24.32(86)	0.182(29)	1.27(29)	4.22(29)	12.87(3)
CP-054	11.52(25)	3.36(9)	0.808(18)	1.355(32)	41.22(50)	0.708(20)	8.16(49)	0.39(11)	2.77(10)	6.22(11)	7.45(20)
CP-021	10.35(31)	5.936(3)	0.487(5)	1.293(35)	22.31(22)	0.426(5)	4.41(22)	0.62(5)	4.40(4)	7.83(5)	8.14(17)
CP-027	16.22(10)	3.368(8)	0.904(28)	1.186(36)	64.95(74)	0.792(31)	12.85(73)	0.55(6)	3.91(5)	7.39(6)	9.79(7)
MTA-22	8.48(46)	2.8(19)	0.764(14)	2.318(16)	28.69(30)	0.669(16)	5.68(30)	0.242(22)	1.70(22)	4.87(22)	5.64(35)
CP-036	16.62(8)	0(53)	1.14(53)	0(53)	83.98(84)	1(53)	16.62(83)	0(53)	0(53)	0(53)	8.31(16)
CP-026	16.72(7)	1.52(35)	1.037(45)	0.638(45)	76.79(82)	0.909(45)	15.20(81)	0.259(18)	1.82(18)	5.04(18)	9.12(11)
CP-025	2.21(86)	0.656(47)	0.802(17)	2.086(19)	7.85(3)	0.702(19)	1.56(3)	0.014(51)	0.10(51)	1.20(51)	1.43(86)
CP-069	8.38(47)	0.992(43)	1.006(43)	0.831(43)	37.34(45)	0.882(43)	7.39(44)	0.085(38)	0.59(38)	2.88(38)	4.69(48)
CP-029	8.24(51)	0(53)	1.14(53)	0(53)	41.63(51)	1(53)	8.24(50)	0(53)	0(53)	0(53)	4.12(58)
CP-049	5.91(71)	4.36(5)	0.299(3)	5.179(3)	7.84(2)	0.262(3)	1.55(2)	0.263(17)	1.84(17)	5.07(17)	5.13(39)
CP-008	6.40(62)	1.8(29)	0.820(21)	1.975(23)	23.24(25)	0.718(23)	4.60(25)	0.117(34)	0.82(34)	3.39(34)	4.10(60)
CP-048	6.51(61)	3.016(15)	0.612(7)	3.252(7)	17.66(14)	0.536(7)	3.49(14)	0.200(27)	1.40(27)	4.43(27)	4.76(47)
CP-068	6.72(59)	2.968(16)	0.977(36)	3.102(9)	18.95(15)	0.558(9)	3.75(15)	0.203(25)	1.43(25)	4.46(25)	4.84(45)
CP-016	18.72(6)	14.77(1)	0.24(2)	5.543(2)	19.92(17)	0.211(2)	3.94(17)	2.82(1)	19.83(1)	16.63(1)	16.74(2)
CP-031	3.26(82)	0(53)	1.14(53)	0(53)	16.49(13)	1(53)	3.26(13)	0(53)	0(53)	0(53)	1.63(83)
CP-028	8.30(49)	3.24(14)	0.696(11)	2.740(12)	25.58(27)	0.609(12)	5.06(27)	0.27(16)	1.92(16)	5.18(16)	5.77(33)
CP-024	8.24(52)	0.648(48)	1.052(47)	0.552(47)	38.35(46)	0.921(47)	7.59(45)	0.054(46)	0.38(46)	2.31(46)	4.44(54)
C	4.00(78)	0(53)	1.14(53)	0(53)	20.20(18)	1(53)	4.00(18)	0(53)	0(53)	0(53)	2.00(81)
D	6.07(69)	0(53)	1.14(53)	0(53)	30.67(33)	1(53)	6.07(33)	0(53)	0(53)	0(53)	3.03(74)
NDT-003	3.81(79)	0(53)	1.14(53)	0(53)	19.23(16)	1(53)	3.80(16)	0(53)	0(53)	0(53)	1.90(82)
F	6.22(68)	0(53)	1.14(53)	0(53)	31.44(34)	1(53)	6.22(34)	0(53)	0(53)	0(53)	3.11(73)
B	6.24(67)	0(53)	1.14(53)	0(53)	31.53(36)	1(53)	6.25(36)	0(53)	0(53)	0(53)	3.12(72)

NDT-010	14.98(14)	3.5(7)	0.874(25)	1.641(28)	57.97(69)	0.766(28)	11.47(68)	0.53(8)	3.75(7)	7.23(8)	9.23(9)
NDT-016	3.62(80)	1.616(34)	0.631(8)	3.138(8)	10.10(7)	0.553(8)	2.00(7)	0.059(45)	0.41(45)	2.41(45)	2.61(78)
NDT-045	6.80(58)	1.144(39)	0.949(34)	1.181(38)	28.57(29)	0.831(38)	5.65(29)	0.079(40)	0.55(40)	2.78(40)	3.97(63)
NDT-004	10.90(29)	1.392(36)	0.995(42)	0.897(42)	48.01(60)	0.872(42)	9.50(59)	0.154(32)	1.08(32)	3.89(32)	6.14(30)
NDT-002	14.88(16)	0(53)	1.14(53)	0(53)	75.17(80)	1(53)	14.88(79)	0(53)	0(53)	0(53)	7.44(21)
E	14.98(15)	0(53)	1.14(53)	0(53)	75.66(81)	1(53)	14.97(80)	0(53)	0(53)	0(53)	7.48(19)
C-9	42.63(1)	1.776(30)	1.094(51)	0.292(51)	206.41(88)	0.958(51)	40.85(87)	0.77(4)	5.42(3)	8.70(4)	22.20(1)
C-7	23.38(3)	1.064(41)	1.089(50)	0.319(50)	112.72(86)	0.954(50)	22.31(85)	0.253(19)	1.78(19)	4.98(19)	12.22(5)
NDT-029	3.20(83)	1.304(38)	0.676(10)	2.862(11)	9.57(5)	0.592(11)	1.89(5)	0.042(47)	0.29(47)	2.04(47)	2.25(80)
C-14	10.18(33)	0(53)	1.14(53)	0(53)	51.45(64)	1(53)	10.18(63)	0(53)	0(53)	0(53)	5.09(43)
C-10	16.36(9)	1.704(33)	1.022(44)	0.731(40)	74.04(79)	0.895(44)	14.65(78)	0.28(15)	1.99(15)	5.27(15)	9.03(12)
C-18	13.52(20)	0(53)	1.14(53)	0(53)	68.30(76)	1(53)	13.52(75)	0(53)	0(53)	0(53)	6.76(25)
C-19	9.60(36)	0(53)	1.14(53)	0(53)	48.50(61)	1(53)	9.60(60)	0(53)	0(53)	0(53)	4.80(46)
NDT-015	13.28(21)	0(53)	1.14(53)	0(53)	67.09(75)	1(53)	13.28(74)	0(53)	0(53)	0(53)	6.64(26)
NDP-017	9.36(40)	0(53)	1.14(53)	0(53)	47.29(59)	1(53)	9.37(58)	0(53)	0(53)	0(53)	4.67(50)
NDT-005	9.92(34)	0(53)	1.14(53)	0(53)	50.11(62)	1(53)	9.92(61)	0(53)	0(53)	0(53)	4.96(44)
NDT-006	5.61(73)	3.288(12)	0.472(4)	4.117(4)	11.72(10)	0.413(4)	2.32(10)	0.188(28)	1.32(28)	4.29(28)	4.45(53)
NDT-025	8.80(44)	0(53)	1.14(53)	0(53)	44.45(54)	1(53)	8.80(53)	0(53)	0(53)	0(53)	4.40(55)
NDT-001	2.56(85)	0(53)	1.14(53)	0(53)	12.93(11)	1(53)	2.56(11)	0(53)	0(53)	0(53)	1.28(87)
A	7.50(56)	1(42)	0.989(39)	0.935(40)	32.85(41)	0.866(40)	6.50(41)	0.076(41)	0.53(41)	2.73(41)	4.25(56)
NDT-009	12.21(22)	0(53)	1.14(53)	0(53)	61.67(71)	1(53)	12.20(70)	0(53)	0(53)	0(53)	6.10(31)
NDT-027	4.96(75)	0.64(50)	0.994(41)	0.906(41)	21.83(20)	0.871(41)	4.32(20)	0.032(49)	0.22(49)	1.78(49)	2.80(76)
NDT-011	2.08(87)	0.85(44)	0.673(9)	2.879(10)	6.20(1)	0.59(10)	1.22(1)	0.018(50)	0.12(50)	1.33(50)	1.46(85)
NDT-008	11.98(23)	2.024(23)	0.948(33)	1.186(36)	50.32(63)	0.831(36)	9.96(62)	0.247(20)	1.73(20)	4.92(20)	7.00(24)
C-8	6.59(60)	0(53)	1.14(53)	0(53)	33.30(42)	1(53)	6.59(41)	0(53)	0(53)	0(53)	3.29(68)
NDT-013	16.16(11)	0(53)	1.14(53)	0(53)	81.64(83)	1(53)	16.16(82)	0(53)	0(53)	0(53)	8.08(18)
NDT-022	3.54(81)	1.84(28)	0.547(6)	3.654(6)	8.56(4)	0.479(6)	1.69(4)	0.066(42)	0.46(42)	2.55(42)	2.68(77)
NDT-018	4.80(76)	1.768(31)	0.721(12)	2.586(13)	15.31(12)	0.631(13)	3.03(12)	0.086(37)	0.60(37)	2.91(37)	3.28(69)
NDT-026	9.10(41)	0(53)	1.14(53)	0(53)	45.99(57)	1(53)	9.10(56)	0(53)	0(53)	0(53)	4.55(51)
NDT-020	6.32(63)	0.504(51)	1.051(46)	0.560(46)	29.38(32)	0.920(46)	5.81(32)	0.033(48)	0.24(48)	1.79(48)	3.41(66)
NDT-023	7.89(54)	0(53)	1.14(53)	0(53)	39.85(48)	1(53)	7.88(47)	0(53)	0(53)	0(53)	3.94(64)
NDT-012	5.79(72)	0(53)	1.14(53)	0(53)	29.26(31)	1(53)	5.79(31)	0(53)	0(53)	0(53)	2.89(75)
NDT-025 B	6.32(64)	0(53)	1.14(53)	0(53)	31.93(37)	1(53)	6.32(37)	0(53)	0(53)	0(53)	3.16(70)

Values in the parentheses refer to the ranks of the genotype for each index, YSi2= Mean value of grain yield under drought stress condition, Ypi=mean value of yield under non-stress condition, SSI= Stress susceptibility index, RDI=relative drought index, SSPI= Stress susceptibility percentage index, SDI= Sensitivity drought index, TOL= stress tolerance, STI= Stress tolerance index, REI= The relative efficiency index, GMP= Geometric mean productivity, MP= Mean productivity, YSI= Yield stability index, YI= Yield index, PYR= percentage of yield reduction, DTI= Drought tolerance index, HM= Harmonic mean, DII = Drought Intensity Index

Correlations between drought tolerant indices and seed yield

To determine the most desirable drought tolerant selection criteria, Pearson correlation coefficients between Yp, Ys, and the fifteen different indices related to drought tolerance were estimated and are presented in Table 5. Under both stress at the vegetative (VDS) and flowering setting stage (FDS), there were positive and significant correlation between Yp and Ys: r = 0.36 and 0.22 for VDS and FDS respectively. Grain yield under both stress and non-stress conditions significantly and positively correlated with STI, REI, GMP, MP, YI and HM at both VDS and FDS (Table 5). For both VDS and FDS, mean grain yields under stresses conditions (Ys) were significantly and positively correlated with RDI, STI, REI, GMP, MP, YSI, YI, DTI, and HM; significantly and negatively associated with these four indices namely SSI, SDI, PYR and DII (Table 5)

Table 5: Pearson correlation coefficients between fourteen drought tolerance indices and cowpea grain yield for drought stress at the vegetative stage (lower diagonal) and drought stress at the flowering setting stage (upper diagonal).

Variables	Ypi	Ysi_2	SSI	RDI	SSPI	SDI	TOL	STI	REI	GMP	MP	YSI	YI	PYR	DTI	HM	DII
Ypi		**0.22**	0.12	-0.13	**0.94**	0.13	**0.94**	**0.43**	**0.43**	**0.44**	**0.95**	-0.13	**0.22**	0.13	0.11	**0.27**	0.13
Ysi_1	**0.36**		**-0.80**	**0.81**	-0.15	**-0.81**	-0.15	**0.92**	**0.92**	**0.91**	**0.51**	**0.81**	**1.00**	**-0.81**	**0.92**	**0.98**	**-0.81**
SSI	0.12	**-0.79**		**-0.98**	**0.42**	**0.98**	**0.42**	**-0.57**	**-0.57**	**-0.69**	-0.15	**-0.98**	**-0.80**	**0.98**	**-0.74**	**-0.78**	**0.98**
RDI	-0.14	**0.78**	**-0.98**		**-0.43**	**-1.00**	**-0.43**	**0.57**	**0.57**	**0.70**	0.14	**1.00**	**0.81**	**-1.00**	**0.74**	**0.80**	**-1.00**
SSPI	**0.89**	-0.11	**0.52**	**-0.53**		**0.43**	**1.00**	0.10	0.10	0.11	**0.78**	**-0.43**	-0.15	**0.43**	**-0.23**	-0.08	**0.43**
SDI	0.14	**-0.78**	**0.98**	**-1.00**	**0.53**		**0.43**	**-0.57**	**-0.57**	**-0.70**	-0.14	**-1.00**	**-0.81**	**1.00**	**-0.74**	**-0.80**	**1.00**
TOL	**0.89**	-0.11	**0.52**	**-0.53**	**1.00**	**0.53**		0.10	0.10	0.11	**0.78**	**-0.43**	-0.15	**0.43**	**-0.23**	-0.08	**0.43**
STI	**0.71**	**0.80**	**-0.36**	**0.35**	**0.37**	**-0.35**	**0.37**		**1.00**	**0.87**	**0.67**	**0.57**	**0.92**	**-0.57**	**0.87**	**0.90**	**-0.57**
REI	**0.71**	**0.80**	**-0.36**	**0.35**	**0.37**	**-0.35**	**0.37**	**1.00**		**0.87**	**0.67**	**0.57**	**0.92**	**-0.57**	**0.87**	**0.90**	**-0.57**
GMP	**0.63**	**0.91**	**-0.59**	**0.58**	**0.23**	**-0.58**	**0.23**	**0.89**	**0.89**		**0.67**	**0.70**	**0.91**	**-0.70**	**0.71**	**0.96**	**-0.70**
MP	**0.94**	**0.66**	-0.19	0.17	**0.68**	-0.17	**0.68**	**0.87**	**0.87**	**0.85**		0.14	**0.51**	-0.14	**0.39**	**0.55**	-0.14
YSI	-0.14	**0.78**	**-0.98**	**1.00**	**-0.53**	**-1.00**	**-0.53**	**0.35**	**0.35**	**0.58**	0.17		**0.81**	**-1.00**	**0.74**	**0.80**	**-1.00**
YI	**0.36**	**1.00**	**-0.79**	**0.78**	-0.11	**-0.78**	-0.11	**0.80**	**0.80**	**0.91**	**0.66**	**0.78**		**-0.81**	**0.92**	**0.98**	**-0.81**
PYR	0.14	**-0.78**	**0.98**	**-1.00**	**0.53**	**1.00**	**0.53**	**-0.35**	**-0.35**	**-0.58**	-0.17	**-1.00**	**-0.78**		**-0.74**	**-0.80**	**1.00**
DTI	0.08	**0.90**	**-0.84**	**0.83**	**-0.35**	**-0.83**	**-0.35**	**0.59**	**0.59**	**0.67**	**0.40**	**0.83**	**0.90**	**-0.83**		**0.83**	**-0.74**
HM	**0.49**	**0.98**	**-0.71**	**0.70**	0.05	**-0.70**	0.05	**0.86**	**0.86**	**0.97**	**0.75**	**0.70**	**0.98**	**-0.70**	**0.79**		**-0.80**
DII	0.14	**-0.78**	**0.98**	**-1.00**	**0.53**	**1.00**	**0.53**	**-0.35**	**-0.35**	**-0.58**	-0.17	**-1.00**	**-0.78**	**1.00**	**-0.83**	**-0.70**	

Values in Bold are Significant at 0.050 probability level.

Principal component analysis

Principal component analysis (PCA) revealed that under VDS, the first 2 principal components, PC1 and PC2 explained respectively 61.44 and 33.13% (both 94.57%) of the total variation (Fig. 1). The first two components under FDS corresponded to the essential variance in cowpea genotypes, accounting for 93.42% cumulatively (PC1=64.44% and PC2=33.13%, Fig. 2). The indices STI, REI, GMP, MP, YI, HM, Ys, and Yp contributed positively and significantly to PC1 for both stresses. These indices were strongly associated with yield under stress and non-stress conditions and therefore, found as the most suitable indices to screen high-yielding and drought-tolerant genotypes under both stress and non-stress conditions. Accordingly, this first component could be named component of yield potential and tolerance to drought stress and associated genotypes from the biplot analysis are to be high yielding under non-stress and stress conditions. As a result, genotypes CP-016, C-9, MTA-22, CP-046, CP-060 for VDS phase and CP-016, CP-056, CP-021, CP-049, CP-040, NDT-006 for FDS stage with the higher and significant PC1 values and valueless PC2 were superior plants under both non-stress and moisture stress conditions (Fig. 1 and 2). Meanwhile, under the two moisture stress regimes, Genotypes with larger PC2 and valueless PC1 scores performed poorly. These were CP-004, C7, CP-042, CP-034, CP-031 and CP-019 for VDS stage and C-9, C-7, CP-004, NDT-011, NDT-006, CP-034 and CP-031 for FDS stage (Fig. 1 and 2). Considering Ys, the best index revealed by the biplot analysis was YI as their association was strong and positive, showing genotypes CP-016, C9, CP-014, MTA-22 under VDS (Fig. 1) and CP-016, CP056, CP-021, CP-042 under FDS (Fig. 2) as best suited in stress environments. In contrast, large obtuse angles between the vectors of SSI, SDI, and PRY indices with Ys, indicated a strong negative association between those indices and Ys. The bi-plot displayed a series of genotypes with valueless Ys (like CP-037, NDT-013, CP-054, CP-003, CP-067, CP-020) as highly drought sensitive with low yield stability. Biplots

analysis also revealed a strong negative association between SSI, DTI, and PYR on one hand with YSI and RDI values on the other hand. The highest YSI and RDI values were obtained by genotypes CP-046 MTA-22, CP-060, D, CP-068, CP-016 and CP-033 under VDS and CP-046, CP-016, CP-049, NDT-006 under FDS, showing high grain yield stability and important tolerance ranking.

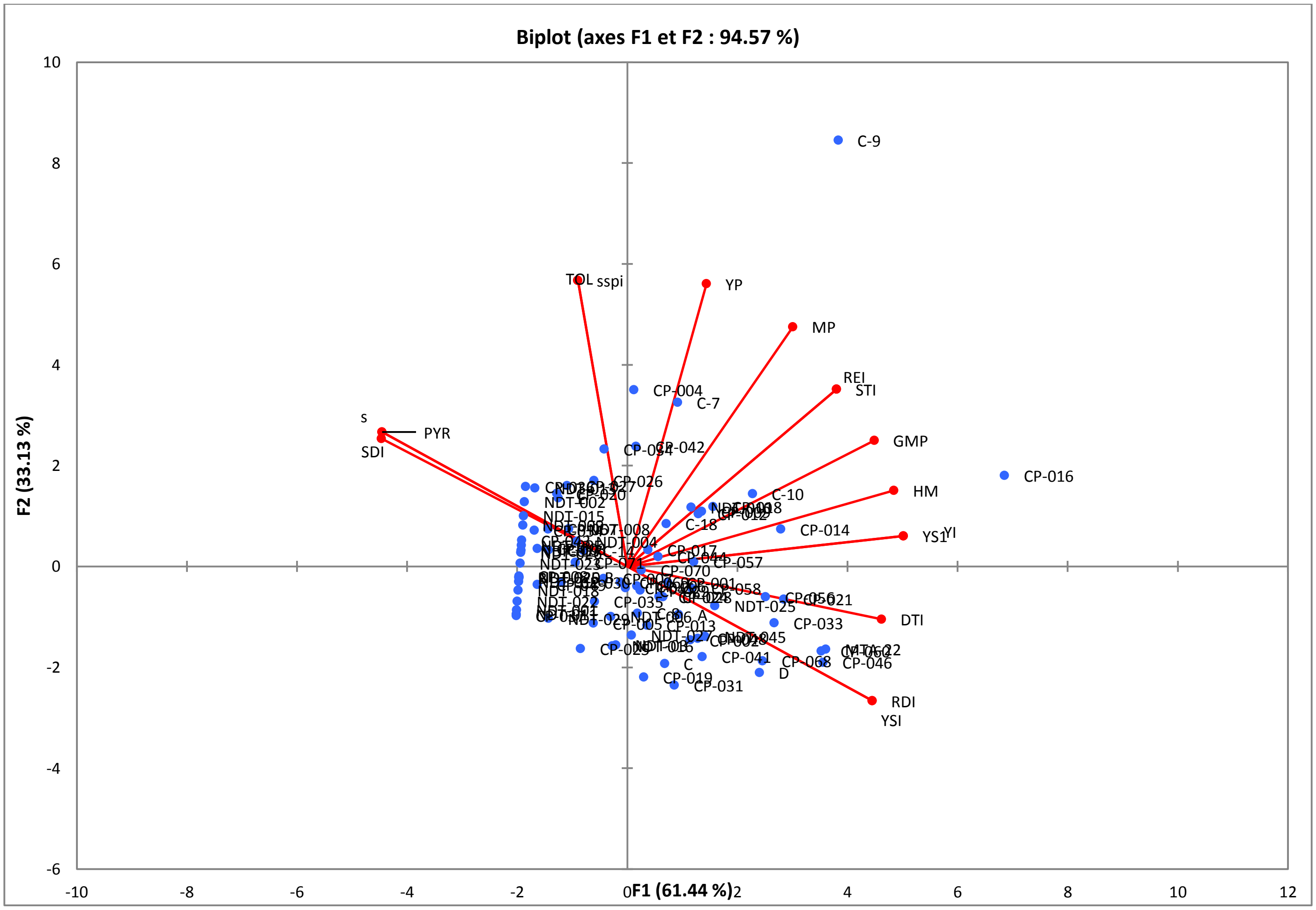

Fig. 1: Biplot for drought tolerance indices based on the first two principal components axes (PC1 and PC2) for eighty-eight cowpea genotypes under VDS

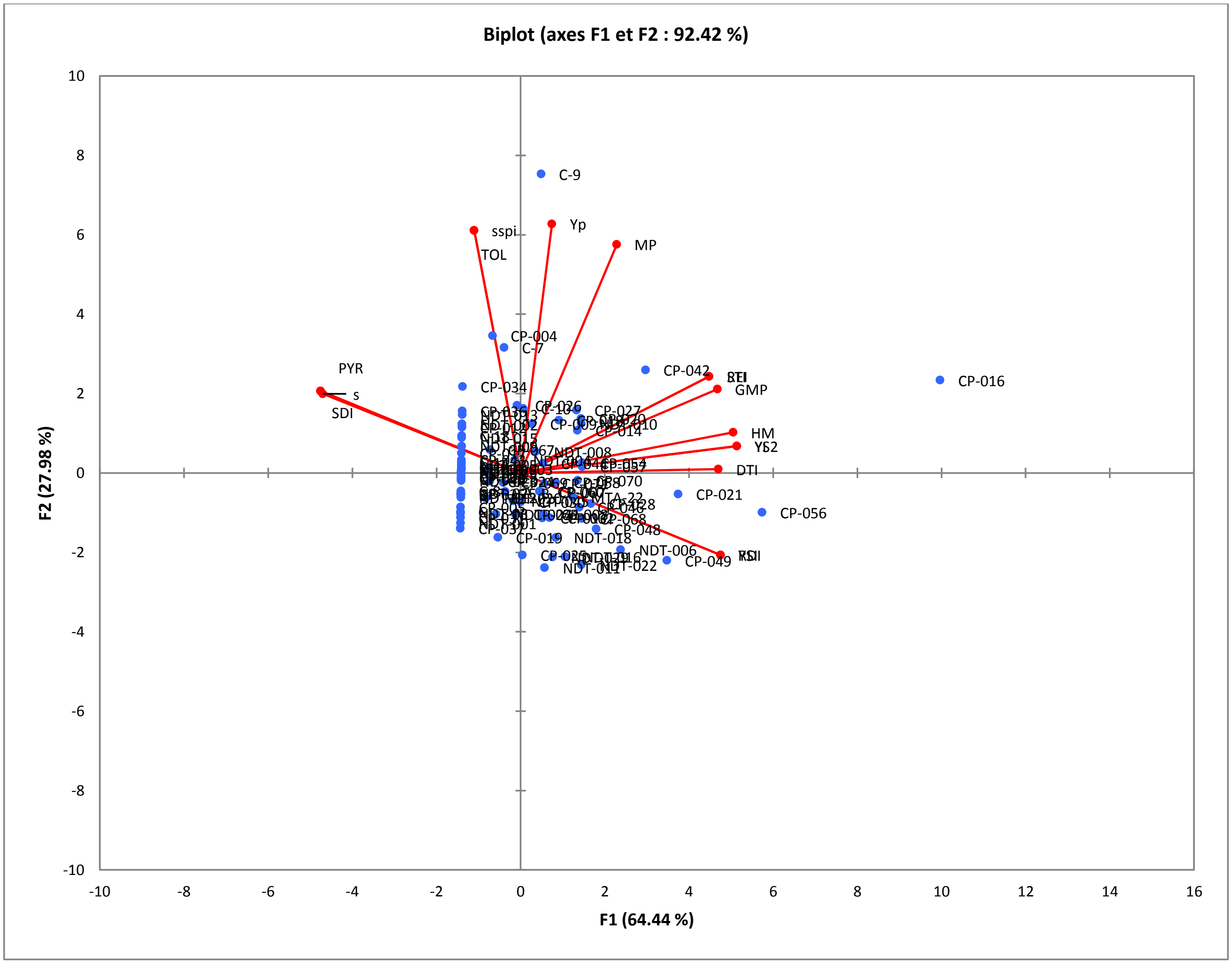

Fig. 2: Biplot for drought tolerance indices based on the first two principal components axes (PC1 and PC2) for eighty-eight cowpea genotypes under FDS

Cluster analysis

Cluster analysis based on fifteen drought tolerance indices and grain yield under VDS and FDS conditions produced each a dendrogram that divided the eighty-eight cowpea genotypes in three main clusters. Under VDS (Fig. 3), cluster 1 was the largest, containing 51 genotypes (57.95%) followed by cluster 3, which contained 35 genotypes (39.77%) and cluster 2 was the smallest group containing

two genotypes (2.27%). While under FDS, cluster 3 was the largest, containing 51 genotypes (57.95%) followed by cluster 1, with 36 genotypes (41%) and cluster 2 consisted of a single genotype (1.13%) (Fig. 4). Genotypes grouped in cluster 1 for both VDS and FDS were characterized in general by highest YSI, DRI, Ys, and YI indices which was considered a stable group under both stress and non-stress conditions. Genotypes grouped in this cluster were identified as tolerant or semi-tolerant to moisture stress. From this group, subgroups have been distinguished, among which genotypes with moderate to high grain yield with high stability under both moisture stress conditions like CP-046, MTA-22, CP-060, D for VDS and CP-056, CP-021, CP-049 under FDS. Another sub-group clustered genotypes with least stability performance like CP-034, CP-002, C-10, CP-014 for VDS and MTA-22, CP-026 under FDS. Under VDS, cluster 2 had two genotypes (CP-016, C-9) and CP016 was the only genotype for Cluster 2 under FDS. These genotypes, principally CP-016 had higher performance in both stressed and non-stressed environments than any other genotypes tested in this study. Moreover, these genotypes had high grain yield under both moisture regimes as well as reliable drought tolerance indices such as STI, REI, GMP, MP, YI, DTI, HM and HM. Therefore, this group was considered as having the most desirable genotype for both growth conditions and tolerant to moisture stress. Genotypes grouped in cluster 3 were characterized by poor-yield performance under moisture stress conditions. From this clusters, two others subgroups were identified. The first sub-groups clustered for example NDT-013, CP-036, NDT-015 and NDT-009 for VDS, C-9, CP-004, C-7 and CP-034 for FDS. These genotypes were characterized by high to moderate performance under non-moisture stress conditions and had high values of SSI, TOL, and SSPI indices. They were susceptible to drought and suitable for non-stress conditions only. Under VDS, genotypes named CP-037, NDT-001, NDT-011, NDT-029 and CP-019, CP-025, CP-029, NDT-009 under FDS were clustered together in one subgroup and had the lowest values for STI, HM, GMP, MP, Ys, and Yp.

Hence, these genotypes performed poorly under both stress and non-stress conditions and were also identified as highly sensitive genotypes to drought with least stability performance

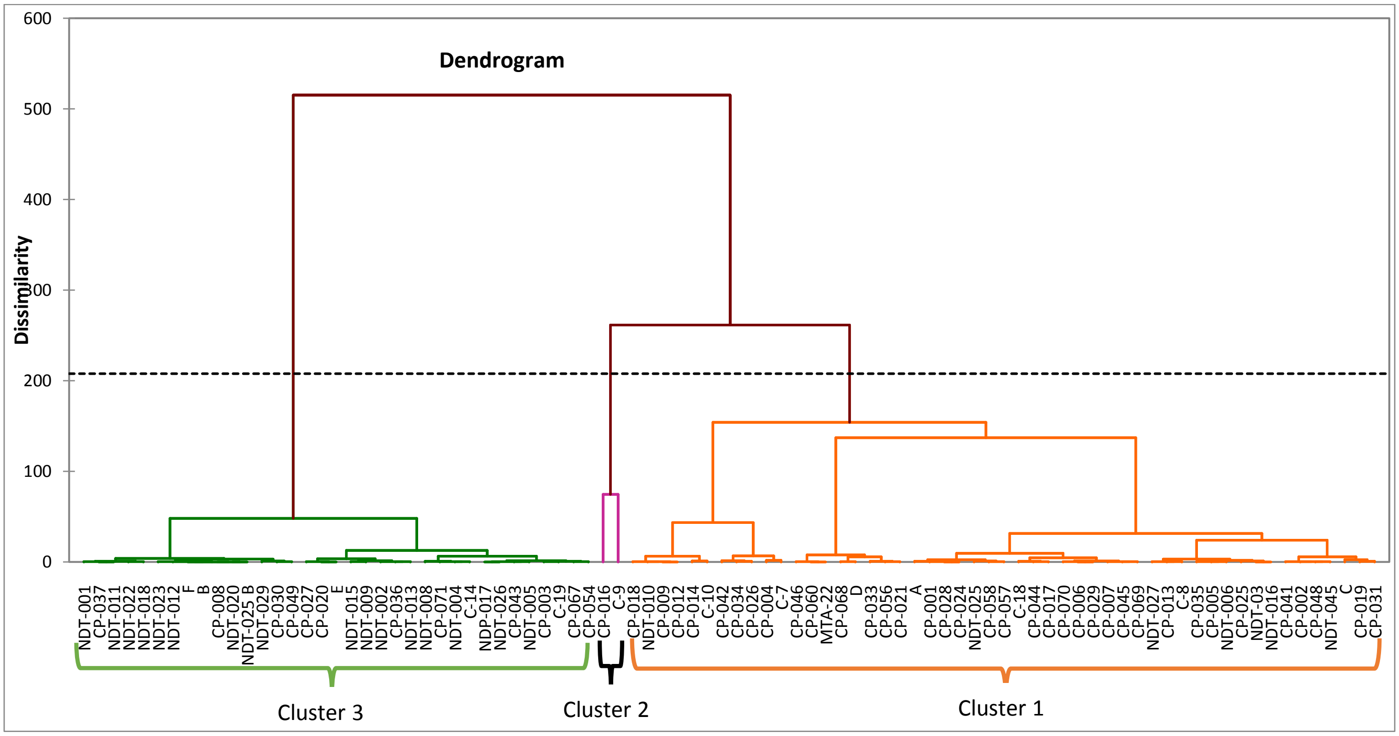

Fig. 3: Dendrogram grouping the eighty-eight cowpea genotypes using average linkage method based on fifteen drought tolerance indices under VDS.

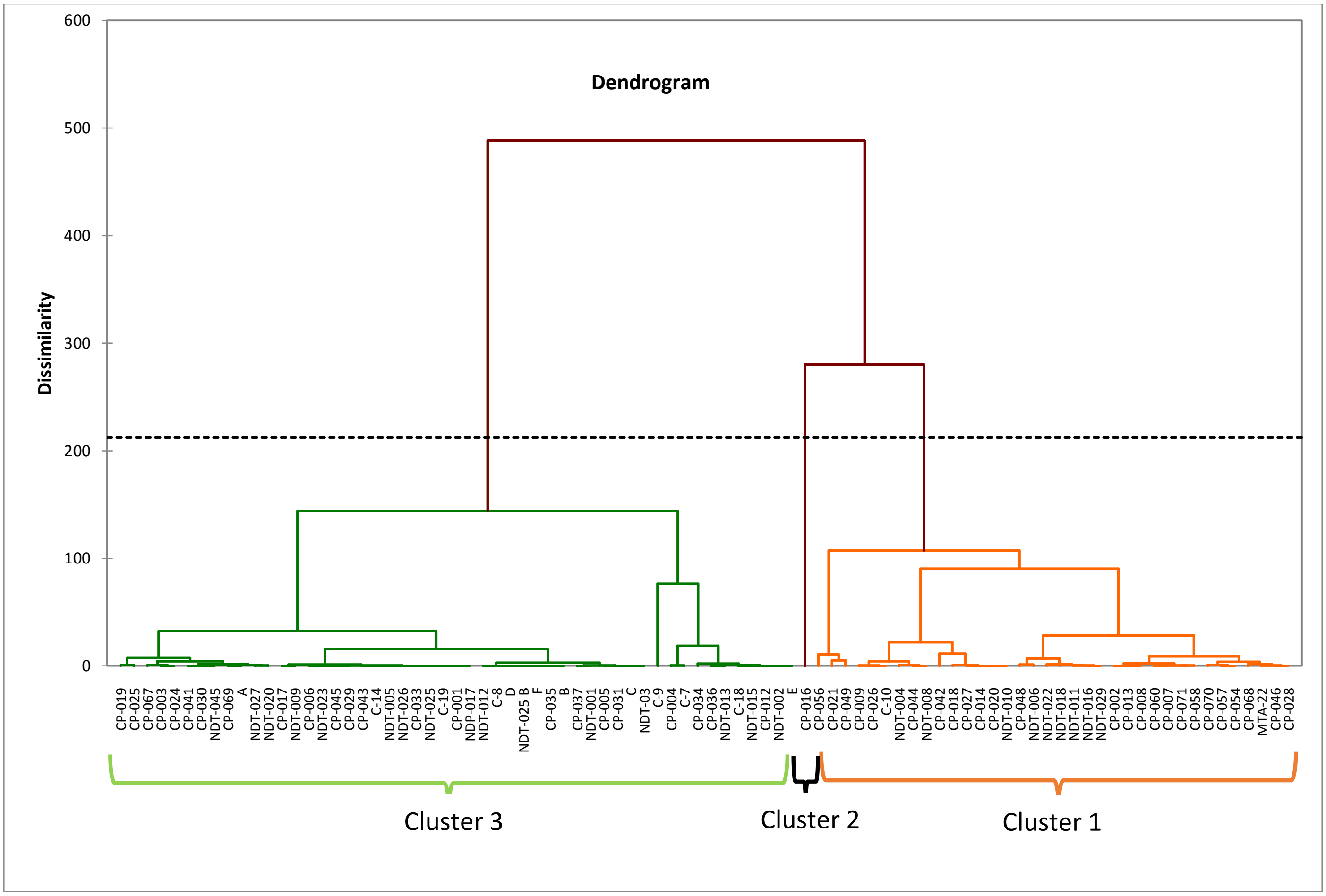

Fig. 4: Dendrogram grouping the eighty-eight cowpea genotypes using average linkage method based on fifteen drought tolerance indices under FDS.

Discussion

According to the literature, moderate to high stress levels are useful and considered ideal for genotypic selection as insufficient stress could result in selection of non-resistant genotypes [48, 49]. Cowpea genotypes in this study were evaluated under severe drought stress as shown a drought intensity index of 0.71 and 0.84 respectively for drought stress initiated at the vegetative and flowering setting stages. These high indices revealed the significant impact of drought applied at both

stress stages compared non-stress conditions. Both drought stresses were suitable for this study, revealing genotypic differences as seen by differential responses for yield in the studied genotypes. This is agreement with the study of Pejic et al. [22] on cowpea and Darkwa et al. [31] in common bean who also reported genotypic variation in their studied accessions under drought stress. This drought severity found led to a significant reduction in yields from unstressed plants: 70.82 and 83.65% respectively for stress during the vegetative and flowering setting stages. Ambachew et al. [25] and Beebe et al. [50] reported similar results when studying common bean with drought stress reducing seed yield to very low levels. Although both high, DII was significantly high under FDS compared to VDS. The same trend was observed for SDI, SSPI and PYR with values under FDS significantly higher compared to values obtained under VDS. This result reveals the sensitiveness of the flowering stage to drought compared to the vegetative stage and confirms previous reports on cowpea by Turk et al. [21] and wheat by Bahar and Yildirim [51] who showed that drought stress negatively and significantly affects flowering and grain filling with considerable yield reduction. Lisar et al. [52] noted that the yield reduction was 50% lower than the control plants for accessions under water stress during the setting of flowers. Oury et al. [53] also observed that water deficit conditions are responsible for large number of flower abortions with subsequent reduction of crop yield. The severity of this stress (FDS) compared to VDS is because it affects parameters related to seed formation including photosynthesis and amino acid metabolism [54, 55].

Understanding of the relationships among yield-based indices in connection to drought tolerance under drought-stress conditions provides to plant breeders desirable drought tolerant selection criteria. Yield under NDS (Yp) and yield under drought stress conditions (Ys) were positively and significantly correlated, indicating the possible indirect selection of genotypes under drought stress condition to be done based on their performance in non-stress conditions. This positive and

significant relationship was also reported by Fernandez [38], Farshadfar and Elyasi [56] and Ferede et al. [57]. Significant correlation between grain yields under moisture conditions (Ys) with some indices indicated the useful of these indices for selection of genotypes expressing uniform and high grain yield under drought stress and non-stress conditions. Significant association were found between many couple of drought tolerance indices (Table 5). We found in both VDS and FDS significant and positive correlation between mean grain yields under stress (Ys) with some indices on the one hand and significant and negative correlation recorded between Ys and some others stress indices on the other hand (Table 5). This is indicative that selection for some low indices value enable selection for high yield under drought stressed condition. These observations are similar to those found when studying maize under drought stress by Naghavi et al. [59] and wheat landrace exposed to moisture stress conditions by Farshadfar and Elyasi [56], Moosavi et al. [39]. Susceptibility of cowpea genotypes to drought stress based on a single criterion does not provide conclusive result as different indices may identify different genotypes as drought tolerant. For example: at the vegetative stage, the highest SSPI and TOL showed that C-9 is among the sensitive genotype while according to the STI, GM, MP, YI, REI and HM index, genotype C-9 was considered as one of the drought tolerant genotype. Therefore, the differentiation and separation of genotypes with different levels of drought tolerance was carried out taking into account all tolerance indices as also carried out Pejic et al. [22] in cowpea, Naghavi et al. [58] and Shojaei et al. [34] in maize, Darkwa et al. [31] in common bean and Yahaya et al. [37] in sorghum when differentiating susceptible from tolerant genotypes using yield-based drought tolerant indices.

From STI, REI, GMP, MP, YI and HM indices, biplot diagram of the principal component analysis (PCA) revealed high-yielding and drought tolerant genotypes under both stress and non-stress conditions. Mau et al [59] in rice, Farshadfar and Elyasi [56] in wheat and Naghavi et al. [58] in

maize reported the useful of these indices as tools in selecting drought tolerant and high yielding genotypes. Based on YSI index, some genotypes evaluated to a stresses environments were suited for the stress environment while in contrast, a series of genotypes with low and valueless YSI were highly drought sensitive with low yield stability. Under VDS and FDS, high grain yield stability with moderate to high tolerance was obtained through YSI and RDI indices. Bouslama and Schapaugh [42] also concluded that cultivars with high YSI are expected to have high to moderate yield under stress and non-stress conditions, yield stability being more important than high yield as also concluded Moosavi et al. [39]. Cluster analysis based on the studied yield-based drought tolerance indices and grain yield under VDS and FDS produced each a dendogram with three main groups likely in link with tolerant, semi-tolerant and sensitive genotypes. Genotypes characterized by highest YSI, DRI, Ys, and YI indices were considered a stable group under both conditions. The genotypes in this group will have high stability with moderate to high grain yield (most of genotypes in cluster 1 for both VDS and FDS). They were identified as tolerant or semi-tolerant genotypes to moisture stress. Similarly to our studies, Gavuzzi et al, [44] suggested the yield index (YI) while and Bouslama and Schapaugh [42] suggested the yield stability index (YSI) to evaluate the stability of genotypes in the both stress and non-stress conditions and identified tolerant genotypes. Cluster 2 contains two genotypes (CP-016 and C-9) for VDS and one genotype (CP-016) for FDS. These genotypes had a higher performance in both stress and non-stress environments than any other genotypes tested. Moreover, these genotypes had high grain yield under both moisture regimes as well as reliable drought tolerance indices such as STI, GMP, MP, YI, DTI and HM. Therefore, these genotypes grouped in cluster 2 can be identified as group A of Fernandez [41] and considered as the most desirable cluster with tolerant genotypes to moisture stress. Farshadfar and Elyasi [56] also identifies similar cluster when studying wheat under drought stress. From Poor-yield performance genotypes

under stress and high to moderate performance genotypes under NDS conditions, classified as Group B according to Fernandez [38] had high values of SSI, TOL, and SSPI indices. Thus, they were susceptible to drought and suitable for non-stress conditions only, while the poorest performance genotypes under both stress and non-stress conditions, pooled in group D following Fernandez [38], had the lowest values for STI, HM, GMP, MP, YSI, and Yp. Hence, these genotypes were identified as highly sensitive genotypes to drought with no stability performance. The majority of these genotypes were found in cluster 3 of the dendogram for both VDS and FDS. Identifying sensitive genotypes from clustering accessions by the help of yield-based drought tolerance indices was also performed on *Phaseolus vulgaris* accessions [31] and on *Eragrostis tef* genotypes as reported Ferede et al. [60]

Conclusion

This study showed a decreased in grain yield of 70.50 and 83.60 % respectively when cowpea plants are exposed to drought stress at the vegetative and the flowering setting stages. This severe drought allowed the discrimination of cowpea genotypes in both VDS and FDS. Drought intensity index, Stress susceptibility percentage index, Percentage of yield reduction where significantly lower for VDS compared to FDS and Ys, STI, GMP, MP and DTI were significantly higher for VDS compared to FDS, pointing the drought at the flowering setting stage being significantly more sensitive and impacting negatively seed yield compared to drought that can take place at the vegetative stage. Based on principal component analysis, the indices STI, REI, GMP, MP, YI and HM. exhibited strong correlation with yield under stress and non-stress conditions and are suitable discriminating high-yielding and drought tolerant genotypes under both stress and non-stress conditions. Based on ranking, PCA and cluster analysis of the studied cowpea genotypes, the top five tolerant to drought

stress under VDS were CP-016, CP-021, MTA-22, CP-056 and CP-060 and were CP-016, CP-056, CP-021, CP-028 and MTA-22 under FDS

Availability of data and materials

The data used to support the findings of this research could be obtained from the corresponding author upon request.

Conflict of interest

The authors declare that they have no conflicts of interest regarding the publication of this article.

Funding

This research did not receive any specific grant from funding agencies in the public, commercial, or not-for-profit sectors.

Author contributions

EBK conceived of the study. EBK, TND collected the plant material. TND, RAB performed the green house experimental trial and collected the data. EBK, TND wrote the manuscript and analyzed the data. MSM reviewed the manuscript prior to submission and provided valued comment on the interpretation of results, AHC and HBD contributed to the scientific content and review of the manuscript. All authors have read and accepted the final version of the manuscript.

References

[1] Osipitan OA, Fields JS, Lo S, Cuvaca I. Production Systems and Prospects of Cowpea (*Vigna unguiculata* (L.) Walp.) in the United States. *Agronomy* 2021; 11: 2312. doi: 10.3390/agronomy11112312

[2] Togola A, Datinon B, Laouali A, Traore´ F, Agboton C, Ongom PO, Ojo JA, Pittendrigh B, Boukar O and Tamò M. Recent advances in cowpea IPM in West Africa. Frontiers in Agronomy 2023; 5: 1220387. doi: 10.3389/fagro.2023.1220387

[3] Abebe BK, Alemayehu MT. A review of the nutritional use of cowpea (Vigna unguiculata L. Walp) for human and animal diets. Journal of Agriculture and Food Research 2022; 10: 100383. https://doi.org/10.1016/j.jafr.2022.100383.

[4] Dakora FD, Belane AK (2019). Evaluation of Protein and Micronutrient Levels in Edible Cowpea (Vigna Unguiculata L. Walp.) Leaves and Seeds. Frontiers in Sustainable Food Systems 2019; 3: 70. doi: 10.3389/fsufs.2019.00070

[5] Ezin V, Tosse AGC, Chabi IB, Ahanchede A. Adaptation of cowpea (Vigna unguiculata (L.) Walp.) to water deficit during vegetative and reproductive phases using physiological and agronomic characters. International Journal of Agronomy *2021*; Article ID 9665312 12 pages. https://doi.org/10.1155/2021/9665312

[6] Horst WJ, Härdter R. Rotation of maize with cowpea improves yield and nutrient use of maize compared to maize monocropping in an alfisol in the northern Guinea Savanna of Ghana. *Plant and Soil* 1994; 160: 171–183. https://doi.org/10.1007/BF00010143

[7] Elowad HOA, Hall AE. Influences of early and late nitrogen fertilization on yield and nitrogen fixation of cowpea under well-watered and dry field conditions. *Field Crops Research 1987; 15*: 229-244. https://doi.org/10.1016/0378-4290(87)90012-8

[8] Sibhatu B, Belete K, Tessema T. Effect of cowpea density and nitrogen fertilizer on a sorghum-cowpea intercropping system in Kobo, northern Ethiopia. International Journal of Agriculture and Forestry 2015*; 5:* 305-317. doi:10.5923/j.ijaf.20150506.02

[9] Kyei-Boahen S, Savala CE, Chikoye D, Abaidoo R. (2017). Growth and yield responses of cowpea to inoculation and phosphorus fertilization in different environments. *Frontiers in plant science* 2017; *8*: 646. https://doi.org/10.3389/fpls.2017.00646

[10] Owade JO, Abong' G, Okoth M, Mwang'ombe AW. *A review of the contribution of cowpea leaves to food and nutrition security in East Africa. Food Science and Nutrition 2020; 8: 36–47*. https://doi.org/10.1002/fsn3.1337

[11] Langyintuo AS, Lowenberg-DeBoer J, Faye M, Lambert D, Ibro G, Moussa B, Ntoukam G. Cowpea supply and demand in West and Central Africa. *Field crops research 2003; 82:* 215-231. https://doi.org/10.1016/S0378-4290(03)00039-X

[12] Bidima IM. Haricot niébé: L'or blanc du Sahel. *La voix du Paysan-Mensuel de l'entrepreneur rural 2012*. 14 pages

[13] Dugje IY, Omoigui LO, Ekeleme F, Kamara AY, Ajeigbe H. Production du niébé en Afrique de l'Ouest: Guide du paysan. *IITA 2009 ; Ibadan, Nigeria, 20* pages

[14] Kouam EB, Ngompe-Deffo T, Anoumaa M, Pasquet RS. (2018). Preliminary study on character associations, phenotypic and genotypic divergence for yield and related quantitative traits among cowpea landraces (Vigna unguiculata) from the Western Highland Region of Cameroon. Open Agriculture 2018; 3: 84–97. doi:10.1515/opag-2018-0009

[15] Fahad S, Bajwa AA, Nazir U, Anjum SA, Farooq A, Zohaib A, Sadia S, Nasim W, Adkins S, Saud S, Ihsan MZ, Alharby H, Wu C, Wang D and Huang J. Crop Production under Drought and Heat Stress: Plant Responses and Management Options. Frontiers in plant sciences 2017; 8: 1147. https://doi.org/10.3389/fpls.2017.01147

[16] Seleiman MF, Al-Suhaibani N, Ali N, Akmal M, Alotaibi M, Refay Y, Dindaroglu T, Abdul-Wajid HH, Battaglia ML. Drought Stress Impacts on Plants and Different Approaches to Alleviate Its Adverse Effects. Plants 2021; 10: 259. https://doi.org/10.3390/plants10020259

[17] Ntali YM, Lyimo JG, Dakyaga F. Trends, impacts, and local responses to drought stress in Diamare Division, Northern Cameroon, World Development Sustainability 2023; 2: 100040. https://doi.org/10.1016/j.wds.2022.100040.

[18] Salman SA, Shahid S, Sharafati A, Salem GSA, Bakar AA, Farooque AA, Chung E-S, Ahmed YA, Mikhail B, Yaseen ZM. Projection of Agricultural Water Stress for Climate Change Scenarios: A Regional Case Study of Iraq. *Agriculture* 2021; 11: 1288. https://doi.org/10.3390/agriculture11121288

[19] Wang Z, Xu D, Peng D, Zhang X. (2023). Future climate change would intensify the water resources supply-demand pressure of afforestation in inner Mongolia, China. Journal of Cleaner Production 2023, 407: 137145. https://doi.org/10.1016/j.jclepro.2023.137145.

[20] Santos R, Carvalho M, Rosa E, Carnide V, Castro I. Root and Agro-Morphological Traits Performance in Cowpea under Drought Stress. Agronomy 2020; 10: 1604. https://doi.org/10.3390/agronomy10101604

[21] Turk KJ, Hall AE. (1980). Drought Adaptation of Cowpea. II. Influence of Drought on Plant Water Status and Relations with Seed Yield. Agronomy Journal 1980; *72:* 421-427. https://doi.org/10.2134/agronj1980.00021962007200030005x

[22] Pejić B, Mačkić K, Mikić A, Cupina B, Peksen E, Krstić D, Antanasović S. Effect of water stress on the yield of cowpea (*Vigna unguiculata L. Walp*.) in temperate climatic conditions. Journal of Contemporary Agriculture 2013; 62: 168 – 176.

[23] Benjamin JG, Nielsen DC, Vigil MF. Quantifying effects of soil conditions on plant growth and crop production. Geoderma *2003*; *116:* 137-148. https://doi.org/10.1016/S0016-7061(03)00098-3

[24] Demirevska K, Zasheva D, Dimitrov R, Simova-Stoilova L, Stamenova M, Feller U. Drought stress effects on Rubisco in wheat: changes in the Rubisco large subunit. Acta Physiologiae Plantarum 2009*; 31*: 1129-1138. https://doi.org/10.1007/s11738-009-0331-2

[25] Ambachew D, Mekbib F, Asfaw A, Beebe SE, Blair MW. Trait associations in common bean genotypes grown under drought stress and field infestation by BSM bean fly. The Crop Journal 2015; *3:* 305-316. https://doi.org/10.1016/j.cj.2015.01.006

[26] Thiaw S, Hall AE, Parker DR. (1993). Varietal intercropping and the yields and stability of cowpea production in semiarid Senegal. Field Crops Research 1993; *33*: 217-233. https://doi.org/10.1016/0378-4290(93)90081-W

[27] Bastos EA, Nascimento SPD, Silva EMD, Freire-Filho FR, Gomide RL. Identification of cowpea genotypes for drought tolerance. Revista Ciência Agronômica 2011; *42*: 100-107. https://doi.org/10.1590/S1806-66902011000100013

[28] Nasir MW, Toth Z. Effect of Drought Stress on Potato Production: A Review. Agronomy *2022; 12*: 635. https://doi.org/10.3390/agronomy12030635

[29] Bagheri M, Santos CS, Rubiales D, Vasconcelos MW. (2023). Challenges in pea breeding for tolerance to drought: Status and prospects. Annals of Applied Biology 2023; 183: 108–120. https://doi.org/10.1111/aab.12840

[30] Xinqing S, Kun W, Shi- Kui D, Xiao- Xia H, Mu- Yi K. Regionalisation of suitable herbages for grassland reconstruction in agro- pastoral transition zone of northern China. New Zealand Journal of Agricultural Research 2006; *49:* 73-84. https://doi.org/10.1080/00288233.2006.9513696

[31] Darkwa K, Ambachew D, Mohammed H, Asfaw A, Blair MW. (2016). Evaluation of common bean (Phaseolus vulgaris L.) genotypes for drought stress adaptation in Ethiopia. The Crop Journal 2016, 4: 367–376. https://doi.org/10.1016/j.cj.2016.06.007

[32] Sánchez-Reinoso AD, Ligarreto-Moreno GA, Restrepo-Díaz H. Evaluation of drought indices to identify tolerant genotypes in common bean bush (Phaseolus vulgaris L.). Journal of Integrative Agriculture 2020; 19: 99–107. https://doi.org/10.1016/s2095-3119(19)62620

[33] Wasae A. (2021). Evaluation of Drought Stress Tolerance Based on Selection Indices in Haricot Bean Varieties Exposed to Stress at Different Growth Stages. International Journal of Agronomy 2021; Volume 2021, Article ID 6617874, 9 pages, https://doi.org/10.1155/2021/6617874

[34] Shojaei SH, Mostafavi K, Omrani A, Illés Á, Bojtor C, Omrani S, Mousavi SMN, Nagy J. Comparison of Maize Genotypes Using Drought-Tolerance Indices and Graphical Analysis under Normal and Humidity Stress Conditions. Plants 2022; 11: 942. https://doi.org/10.3390/plants11070942

[35] Khatibi A, Omrani S, Omrani A, Shojaei SH, Mousavi SMN, Illés Á, Bojtor C, Nagy J. Response of Maize Hybrids in Drought-Stress Using Drought Tolerance Indices. *Water* 2022; 14: 1012. https://doi.org/10.3390/w14071012

[36] Fouad HM (2018). Physiological Traits and Drought Tolerance Indices in Advanced Genotypes of Bread Wheat (Triticum aestivum L). Egyptian Journal of Agronomy 2018; 40: 145 – 154. https://doi.org/10.21608/agro.2018.3108.1096

[37] Yahaya MA, Shimelis H, Nebié B, Mashilo J, Pop G. Response of African Sorghum Genotypes for Drought Tolerance under Variable Environments. *Agronomy* 2023; 13: 557. https://doi.org/10.3390/agronomy13020557

[38] Fernandez GCJ. Effective Selection Criteria for Assessing Stress Tolerance. In: Kuo, C.G., Ed., Proceedings of the International Symposium on Adaptation of Vegetables and Other Food Crops in Temperature and Water Stress, AVRDC Publication 1992, Tainan, pp 257-270.

[39] Moosavi S, Yazdi-Samadi B, Naghavi M, Zali A, Dashti H, Pourshahbazi A. Introduction of new indices to identify relative drought tolerance and resistance in wheat genotypes. *Desert* 2007; 12: 165-178. https://doi.org/10.22059/jdesert.2008.27115

[40] Fischer RA, Wood JT. (1979). Drought resistance in spring wheat cultivars. III. Yield associations with morpho-physiological traits. Australian Journal of Agricultural Research 1979, 30: 1001-1020.

[41] Hossain ABS, Sears RG, Cox TS, Paulsen GM. (1990). Desiccation tolerance and its relationship to assimilate partitioning in winter wheat. Crop Science 1990; *30:* 622-627. https://doi.org/10.2135/cropsci1990.0011183X003000030030x

[42] Bouslama M, Schapaugh WTJ. (1984). Stress tolerance in soybeans. I. Evaluation of three screening techniques for heat and drought tolerance. Crop science *1984*; *24:* 933-937. https://doi.org/10.2135/cropsci1984.0011183X002400050026x

[43] Gavuzzi P, Rizza F, Palumbo M, Campanile RG, Ricciardi GL, Borghi B. (1997). Evaluation of field and laboratory predictors of drought and heat tolerance in winter cereals. Canadian Journal of Plant Science 1997; 77: 523-531. https://doi.org/10.4141/P96-130

[44] Rosielle AA, Hamblin J. Theoretical aspects of selection for yield in stress and non-stress environment. Crop science 1981; 21: 943-946. https://doi.org/10.2135/cropsci1981.0011183X002100060033x

[45] Lan J. Comparison of evaluating methods for agronomic drought resistance in crops. *Acta Agriculturae Boreali-occidentalis Sinica 1998*, *7*: 85-87.

[46] Jafari A, Paknejad F, Jami AL-Ahmadi M. Evaluation of selection indices for drought tolerance of corn (Zea mays L.) hybrids. International Journal of plant production 2012; 3: 33-38. https://doi.org/10.22069/ijpp.2012.661

[47] Fischer RA, Maurer R. Drought resistance in spring wheat cultivars. I. Grain yield responses. Australian Journal of Agricultural Research 1978; 29: 897-912. https://doi.org/10.1071/AR9780897

[48] Asfaw A, Blair M. Quantification of drought tolerance in Ethiopian common bean varieties. Agricultural Sciences 2014; 5: 124-139. https://doi.org/10.4236/as.2014.52016.

[49] Rao IM, Beebe SE, Polania J, Ricaurte J, Cajiao C, Garcia R, Rivera M. Can tepary bean be a model for improvement of drought resistance in common bean? African Crop Science Journal 2013*;* 21: 265 – 281

[50] Beebe SE., Rao IM, Cajiao C, Grajales M. Selection for drought resistance in common bean also improves yield in phosphorus limited and favorable environments. Crop Science 2008; 48: 582-592. https://doi.org/10.2135/cropsci2007.07.0404

[51] Bahar B, Yildirim M. Heat and drought resistances criteria in spring bread wheat: Drought resistance parameters. Scientific Research and Essays 2010; *5:* 1742-1745.

[52] Lisar SYS, Motafakkerazad R, Hossain MM, Rahman IMM. (2012) Water Stress in Plants: Causes, Effects and Responses. In: Rahman IMM, Hasegawa H, Eds. Water Stress, InTech, Croatia, 1-14. https://doi.org/10.5772/39363}

[53] Oury V, Tardieu F, Turc O. Ovary Apical Abortion under Water Deficit Is Caused by Changes in Sequential Development of Ovaries and in Silk Growth Rate in Maize. Plant Physiology 2016; 171: 986-96. https://doi.org/10.1104/pp.15.00268.

[54] El-Balla MMA, Hamid AA, Abdelmageed AHA. Effects of time of water stress on flowering, seed yield and seed quality of common onion (Allium cepa L.) under the arid tropical conditions of Sudan. Agricultural Water Management 2013; 121: 149–157. doi:10.1016/j.agwat.2013.02.002

[55] Rering CC, Franco JG, Yeater KM, Mallinger RE. Drought stress alters floral volatiles and reduces floral rewards, pollinator activity, and seed set in a global plant. Ecosphere 2020, 11*:* e03254. https://doi.org/10.1002/ecs2.3254

[56] Farshadfar E, Elyasi P. Screening quantitative indicators of drought tolerance in bread wheat (Triticum aestivum L.) landraces. European Journal of Experimental Biology 2012; 2: 577-584.

[57] Ferede B, Mekbib F, Assefa K, Chanyalew S, Abraha E, Tadele Z. Evaluation of drought tolerance in Tef [Eragrostis tef (Zucc.) trotter] genotypes using drought tolerance indices. Journal of crop science and biotechnology 2020; 23: 107-115. https://doi.org/10.1007/s12892-018-0035-0

[58] Naghavi MR, Aboughadareh AP, Khalili M. Evaluation of drought tolerance indices for screening some of corn (Zea mays L.) cultivars under environmental conditions. Notulae Scientia Biologicae 2013; 5: 388-393. https://doi.org/10.15835/nsb539049

[59] Mau YS, Ndiwa AS, Oematan SS, Markus JE. (2019). Drought tolerance indices for selection of drought tolerant, high yielding upland rice genotypes. Australian Journal of Crop Science 2019; 13: 170-178. https://doi.org/10.3316/informit.338026436411593

[60] Ferede B, Mekbib F, Assefa K, Chanyalew S, Abraha E, Tadele Z. Evaluation of Drought Tolerance in Tef [Eragrostis Tef (Zucc.) Trotter] Genotypes Using Drought Tolerance Indices. Journal of Crop Science and Biotechnology 2020; 23: 107–115. https://doi.org/10.1007/s12892-018-0035-0
