# Supplementary material for: Identifying critical growth stage and resilient genotypes in cowpea under drought stress contributes to enhancing crop tolerance for improvement and adaptation in Cameroon
Source: PLoS One. 2024 Jun 28;19(6):e0304674. doi: 10.1371/journal.pone.0304674 (PMC11213307; doi:10.1371/journal.pone.0304674)
Supplement: Attachment — Submitted filename: Responses to reviewers.docx [file pone.0304674.s003.docx]

**RESPONSES TO REVIEWERS**

5. Review Comments to the Author

Please use the space provided to explain your answers to the questions above. You may also include additional comments for the author, including concerns about dual publication, research ethics, or publication ethics. (Please upload your review as an attachment if it exceeds 20,000 characters)

Reviewer #1: This study on “Yield-based drought tolerance indices in cowpea exposed to stress at vegetative and flowering setting stages identifies sensitive growth phase and drought tolerant genotypes in prospect to crop improvement” reveals very good information about the 14 drought tolerance indices in response to the drought stress under early vegetative stage and flower setting stage of the cow pea genotypes. This study will help in selection of appropriate drought tolerance index based on the requirement. However, the manuscript has a lot of ambiguous sentences. Sentences and grammar need to be improved.

**Sentences and grammar have been improved in the revised manuscript**

Was there any reason for selection of genotypes from Sahelian and western regions of Cameroon?

**We found judicial to include in our study genotypes from these two regions since cowpea are mainly and significantly produced in these both regions**

Table 3 and 4 are too big to have the view. Authors may find more suitable way of presenting the important information of these two tables, and these tables can be kept as supplementary information.

**These Tables 3 and 4 present main results of the manuscript. Please I would like these tables to be present as it is in the manuscript**

In the correlation table 5, different drought tolerance indices behave differently with the yield under VDS and FDS conditions. Authors need to make the interpretations of the results keeping in mind the indices and their components of consideration. Similarly, PCA analyses also gives a very important finding on indices and their association and therefore their appropriate use.

**Thanks for the observations. They were considered in the revised manuscript**

There are some minor improvements needed, and some of which I have mentioned in the attached annotated file.

**All the recommendations have been considered in the revised manuscript**

Reviewer #2: - Please choose the words other than the words present in title and avoid repetition such as word drought.

**Amendment was made on the title in the revised manuscript**

- Please take care of spacing and grammatical errors throughout MS.

**These was done throughout the manuscript**

- I suggest adding a map of the study area and providing more details on climatic conditions.

**The study site was described with geographical coordinates (longitude and latitude), elevation (altitude), average air temperature, relative humidity and soil characteristics in the revised manuscript**

- What is the duration of vegetative and reproductive stage in cowpea? 28 days is not long covering major duration of these stages?

**Life cycle in cowpea is in average 60 days for cowpea from the western region and 40 days for the Sahelian accessions. 28 days (4 weeks) is a quite significant within cowpea life cycle**

o At least temperature data needs to be added in MS.

**Temperature data of the study site is included in the revised manuscript**

o Soil physical properties: particularly texture, field capacity, PAW and PWP must be added. o Soil moisture data needs to be presented to see soil water changes during stress period.

**Details of the soil characteristics have been provided in the revised manuscript**

o How FC was maintained when “Under non-drought stress condition, plants were
regularly watered up to field capacity until physiological maturity.” o 5-liter pot capacity looks insufficient to support growth for 2 plants till harvest. What are the dimensions of pots? Need to see soil moisture data.

**Pots used in the experiment contains holes at the bottom, those pots were place on a plastic plate that received the exceed of water after watering. Then pot was watered until the plastic plate started receiving the exceed of water. This is how the FC was regularly maintained.**

**Pot dimensions were added in the revised manuscript. They were 25X30 cm and were sufficient to contain 2 cowpea plants**

-. ANOVA table must be presented before means.

**ANOVA test was provided in the revised manuscript**

- I suggest replacing Pearson’s table to Correlation plot.

**Correlation table was replaced by correlation plot in the revised manuscript**

- The following articles might be useful in improving introduction and results and discussion.
o https://doi.org/10.3390/plants10122565
o https://doi.org/10.3390/agronomy13092218
o <https://doi.org/10.3390/agronomy12102518>

**These important articles were considered in the revised manuscript**

- Please check and correct references

**References were checked again and corrections were made in the revised manuscript**

Reviewer #3: The manuscript has lacking in leading the reader towards a specific conclusion. So, the direction of the paper or discussion should be oriented towards the objective of the paper.

**As stated in the introduction in the revised manuscript, the paper has three clear distinct objectives and conclusions was made concerning those objectives in the revised manuscript**

-As the paper is completely focused on indices based on yield. So, how the plants responded after imposing the drought treatments is hard to understand only based on yield. Different plants traits during and after treatments, along with their interaction to the grain yield could improve the understanding.

**Thanks so much for the remark. I appreciate it. We will consider it during the next study using these same genotypes. The current research was only focused on yield unde non-stress and stress conditions and drought tolerance indices**

-As the study is based on pot experiment, so how the study results/findings would reflect in the field conditions. Author needs to clarify this.

**This study used a huge quantity of plant material (88 accessions) and needed to be carried out in a controlled environment (Greenhouse) to appreciate the variation in the different irrigation regimes and classify genotypes according to their tolerance to drought that are to be reflected in normal field conditions**

-drought scenarios to the targeted country or region might be included in the Introduction section.

**This have been done in the revised manuscript**
