# Supplementary material for: Identifying critical growth stage and resilient genotypes in cowpea under drought stress contributes to enhancing crop tolerance for improvement and adaptation in Cameroon
Source: PLoS One. 2024 Jun 28;19(6):e0304674. doi: 10.1371/journal.pone.0304674 (PMC11213307; doi:10.1371/journal.pone.0304674)
Supplement: Attachment — Submitted filename: Response to Reviewers.odt [file pone.0304674.s004.odt]

RESPONSES TO REVIEWERS

PONE-D-23-38326R1
Identifying critical growth stage and resilient genotypes in cowpea under drought stress contributes to enhancing crop tolerance for improvement and adaptation in Cameroon
PLOS ONE

Dear Dr. Kouam,

Thank you for submitting your manuscript to PLOS ONE. After careful consideration, we feel that it has merit but does not fully meet PLOS ONE’s publication criteria as it currently stands. Therefore, we invite you to submit a revised version of the manuscript that addresses the points raised during the review process.

==============================
Accepted with minor revision
==============================

6. Review Comments to the Author

Please use the space provided to explain your answers to the questions above. You may also include additional comments for the author, including concerns about dual publication, research ethics, or publication ethics. (Please upload your review as an attachment if it exceeds 20,000 characters)
Reviewer #2: Thank you for revising the manuscript.
Just some quick notes to consider,

Please adjust size of tables 3,4 as the last column is cropped.
Tables 3 and 4 have been adjusted in the revised manuscript. All the columns are now well seen as those tables are now well fit on the pages

I suggest to adjust the axis of PCA to lower range: in this way parameters will be more clear.
The range of the PCA axis was set by the XLSTAT program when performing the analysis
Reviewer #4: Please add PC3 and PC4 also because author calculated only two like PC1 and PC2 so he also calculated latest like PC3 and PC4 with clealy defined
We focussed the principal component analysis on PC1 and PC2 because both explained the essential of the variation that was observed. For VDS PC1 and PC2 cumulated 94.57% of the total variation and the same trend was observed with FDS, PC1 and PC1 cumulating 92.42% of the total variation
